# Modeling the Human Stomach In Vitro: Gastric Organoids in Development, Disease, and Therapy

**DOI:** 10.3390/cells15211973

**Published:** 2026-10-29

**Authors:** Zahra Enteshari Najafabadi, Ireneusz Ryszkiel, Hossein Baharvand, Amir Sadeghi, Agata Stanek, Anna Meyfour

**Affiliations:** 1 Basic and Molecular Epidemiology of Gastrointestinal Disorders Research Center, Research Institute for Gastroenterology and Liver Diseases, Shahid Beheshti University of Medical Sciences, Tehran 16635-148, Iran; enteza96@gmail.com; 2 Department of Internal Medicine, Metabolic Diseases and Angiology, Faculty of Health Sciences in Katowice, Medical University of Silesia in Katowice, 40-635 Katowice, Poland; ryszkielirek@gmail.com; 3 Department of Stem Cells and Developmental Biology, Cell Science Research Center, Royan Institute for Stem Cell Biology and Technology, ACECR, Tehran 16635-148, Iran; h.baharvand@royan-rc.ac.ir; 4 Department of Developmental Biology, School of Basic Sciences and Advanced Technologies in Biology, University of Science and Culture, Tehran 13145-871, Iran; 5 Gastroenterology and Liver Diseases Research Center, Research Institute for Gastroenterology and Liver Diseases, Shahid Beheshti University of Medical Sciences, Tehran 16635-148, Iran; amirsadeghimd@yahoo.com

**Keywords:** organoids, gastric cancer, *Helicobacter pylori*, disease modeling, gastric development, COVID-19

## Abstract

**Highlights:**

**What are the main findings?**

- Gastric organoids accurately model region-specific epithelial differentiation, stem cell niches, and infections like *H. pylori*.
- Emerging organoid-on-a-chip and neuromuscular systems mimic luminal flow and contractions, but fully coordinated motility remains unachieved.

**What are the implications of the main findings?**

- Patient-derived organoids offer strong preclinical platforms for drug testing, though clinical precision medicine needs prospective validation.
- Developing defined, Matrigel-free hydrogels and quality-control standards is essential to translate organoids into clinical therapies.

**Abstract:**

Gastric organoids are advanced three-dimensional models generated from adult or pluripotent stem cells. By reproducing key structural, cellular, and functional features of gastric tissue, they overcome several limitations of conventional two-dimensional cultures and animal models. Gastric organoids provide physiologically relevant platforms for studying stomach development, cellular differentiation, and the molecular mechanisms underlying gastric diseases. This review summarizes the principal cell sources and protocols used to generate gastric organoids and discusses recent advances in culture systems, including two-dimensional organoid-derived monolayers, air–liquid interface cultures, co-culture systems, and organoid-on-a-chip technologies. Particular attention is given to the application of gastric organoids in modeling *Helicobacter pylori* infection, SARS-CoV-2 infection, and gastric cancer, as well as in evaluating therapeutic responses. Their roles in developmental biology, the investigation of congenital disorders, drug screening, potential precision-medicine application, biobanking, and regenerative medicine are also discussed. Despite their considerable potential, current gastric organoid models remain limited: conventional epithelial organoids lack stromal, immune, neural, and vascular components; PSC-derived organoids contain only a limited mesenchymal population; and more complex engineered multicellular tissues still depend largely on in vivo engraftment for maturation. Variable maturation, dependence on poorly defined extracellular matrices, and insufficient standardization further limit their translation.

## 1. Introduction

Although the global incidence of certain gastric diseases, such as peptic ulcers and gastric cancer, has declined in recent years, these diseases continue to impose a substantial burden on healthcare systems in many parts of the world [1,2]. However, the cellular and molecular mechanisms governing stomach development and pathology remain incompletely elucidated. In particular, the regulatory pathways underlying gastric cell differentiation require further investigation [3].

Over the past decades, researchers have relied on conventional in vitro systems such as primary gastric cultures and cell lines to study stomach biology. Despite their widespread use, these models have inherent limitations. These cell lines often fail to accurately recapitulate the stomach’s developmental and physiological processes. In contrast, primary tissue cultures preserve intrinsic cellular heterogeneity but cannot be maintained over extended periods. Moreover, the physiological and structural divergence between animal models and the human stomach limits the translational applications of these models [4]. As a result, developing advanced in vitro models that recapitulate the developmental, functional, and pathological landscape of the human stomach has attracted considerable research interest.

To address these limitations, researchers developed a three-dimensional (3D) culture derived from stem cells, known as gastric organoids. Unlike conventional models, organoids can be maintained for long periods while preserving hallmark features of gastric tissue, such as cellular diversity, functional activity, and region-specific structure. Researchers have applied these systems to investigate stem cell niches, map differentiation pathways, and study cell–cell interactions and regulatory mechanisms. These models are also widely used for modeling gastric cancer, *Helicobacter pylori* (*H. pylori*) infection, and chronic inflammatory disorders, providing experimental platforms that are reproducible and physiologically relevant [5,6].

This review summarizes gastric development and cellular composition and discusses methodologies for generating gastric organoids from adult stem cells (ASCs) and pluripotent stem cells (PSCs). It then highlights recent advances in organoid culture engineering, including the emergence of two-dimensional (2D) organoid systems and the integration of organoid-on-a-chip technologies. Finally, it addresses the diverse applications of gastric organoids in disease modeling, high-throughput drug screening, potential precision-medicine applications, regenerative medicine, and developmental studies. Unlike previous reviews that have primarily focused on specific aspects of gastric organoid generation or selected applications, this review provides comprehensive and comparative information on stem-cell sources, culture platforms, disease-modeling applications, and emerging bioengineering strategies. It also critically highlights the methodological and translational barriers that must be addressed for the reliable implementation of gastric organoids in preclinical research and potential precision-medicine applications. Over the past five years, the field has moved beyond epithelium-only cultures toward three-germ-layer and confinement-based gastric tissues containing smooth muscle and enteric neurons, multi-regional gastric assembloids, bilateral-access gastric organoid-on-a-chip platforms, tissue-derived ECM hydrogels as Matrigel alternatives, formal quality standards for gastric organoids, and prospective co-clinical PDO cohorts (Section 5.2, Section 5.3, Section 5.4, Section 5.5, Section 6.1, Section 6.3 and Section 7). This review evaluates which of these capabilities are established, emerging, or still experimental.

### Literature Search Strategy

To compile this narrative review, we performed a literature search using PubMed and Google Scholar from the earliest available records up to 16 October 2026. Combinations of keywords and MeSH terms were applied using Boolean operators, including (“gastric organoids” OR “stomach organoids”) AND (“adult stem cells” OR “pluripotent stem cells” OR “iPSCs”), (“differentiation” OR “gastric development” OR “parietal cells”), (“culture platforms” OR “air–liquid interface” OR “organoid-on-a-chip” OR "extracellular matrix” OR “Matrigel”), (“*Helicobacter pylori*” OR “SARS-CoV-2” OR “gastric cancer” OR “intestinal metaplasia” OR “*CDH1*”), and ("drug screening” OR “precision medicine” OR “neuromuscular” OR “standardization”). The search was limited to English-language, peer-reviewed primary studies, consensus standards, and clinical trials, excluding conference abstracts and preprints. Relevant review articles and reference lists of retrieved papers were also manually checked to avoid missing key contributions. Studies were prioritized according to their direct relevance to gastric development, organoid culture platforms, disease modeling, and drug evaluation. Because this work was designed as a narrative rather than a systematic review, no formal systematic PRISMA screening or risk-of-bias evaluation was conducted, meaning that certain relevant reports might not have been included.

## 2. Gastric Development and Its Relevance to Organoid Generation

### 2.1. Embryonic Origin of the Stomach

The stomach epithelium originates from the foregut endoderm and begins regionalizing along the proximal–distal axis during development, in close proximity to the esophagus, intestine, and pancreas [7]. Gastric development comprises the formation of definitive endoderm (DE), regionalization of the gut tube, specification of the stomach, and differentiation of its cell types [8]. DE formation is largely controlled by Nodal signaling, a member of the TGF-β family [9]. The primitive gut tube that forms afterwards is patterned along the anterior–posterior axis by FGF, Wnt, retinoic acid (RA), and BMP signaling, and its anterior part, the foregut, gives rise to the esophagus, lungs, liver, pancreas, and stomach, among other organs [10]. Within this tube, *SOX2* and *HHEX* are essential for foregut development, whereas *CDX1* and *CDX2* play a key role in the midgut and hindgut, and the balance between these factors helps to define the boundary between the stomach and the intestine [11,12,13,14].

Gastric development is driven by complex interactions between the epithelium and the mesenchyme, mediated by diverse signaling pathways and transcription factors [9,15]. The stomach originates from three embryonic layers: endoderm (epithelium), mesoderm (smooth muscle and vessels), and neural crest cells (enteric neurons). Communication between these layers, especially epithelial–mesenchymal interactions, is essential for its development [16].

### 2.2. Signaling Pathways That Guide Gastric Identity

Many of the pathways that pattern the foregut and specify the stomach are the same ones that are added to the culture medium to generate gastric organoids. Table 1 summarizes these factors, their role in development, and the stage of the protocols in which they are used. A few of them need further explanation.

BMP signaling negatively regulates SOX2, and suppression of BMP at early stages is essential for the differentiation of esophageal and gastric tissues [24]. This is consistent with the use of Noggin during patterning of DE into posterior foregut [18]. The role of Wnt depends on the region and the stage. Active Wnt signaling is required for the initiation and maintenance of fundic fate, whereas reduced Wnt signaling is critical for stomach specification and for the stomach–intestine boundary, where Barx1 promotes the expression of secreted Wnt antagonists [24]. Organoid studies reflect this difference. Activation of β-catenin with CHIR99021 in foregut progenitors induced the fundic markers *IRX2*, *IRX3*, and *IRX5* and suppressed *PDX1*, an antral marker [18], and in the first mouse antral organoids, reducing WNT concentration induced mucosal and endocrine lineages [19].

### 2.3. Stem Cells and Cell Types of the Adult Gastric Gland

In the adult stomach, the proximal region (corpus and fundus) contains parietal, chief, and mucous cells, while the distal region (antrum) consists primarily of endocrine and mucous cells [24]. Gastric cells differentiate from stem and progenitor cells in the isthmus of the glands, but the position of the isthmus differs between the two regions: it is closer to the surface in fundic glands and nearer to the base in antral glands [7]. In the antrum, Lgr5^+^ cells at the base and Sox2^+^ cells in the isthmus are the two major stem cell populations [19]. Troy^+^ cells at the base of the glands are considered reserve stem cells that are activated in response to tissue damage [25]. Since the glands are enriched in Lgr5^+^ stem cells, they are the starting material for ASC-derived organoids, which are classified as antral or corpus depending on the site of sampling [6]. However, while these organoids faithfully recapitulate epithelial differentiation, standard models lack the non-epithelial mesenchymal, neural, vascular, and immune components derived from the other embryonic germ layers [4,26].

## 3. History

**Figure 1:**
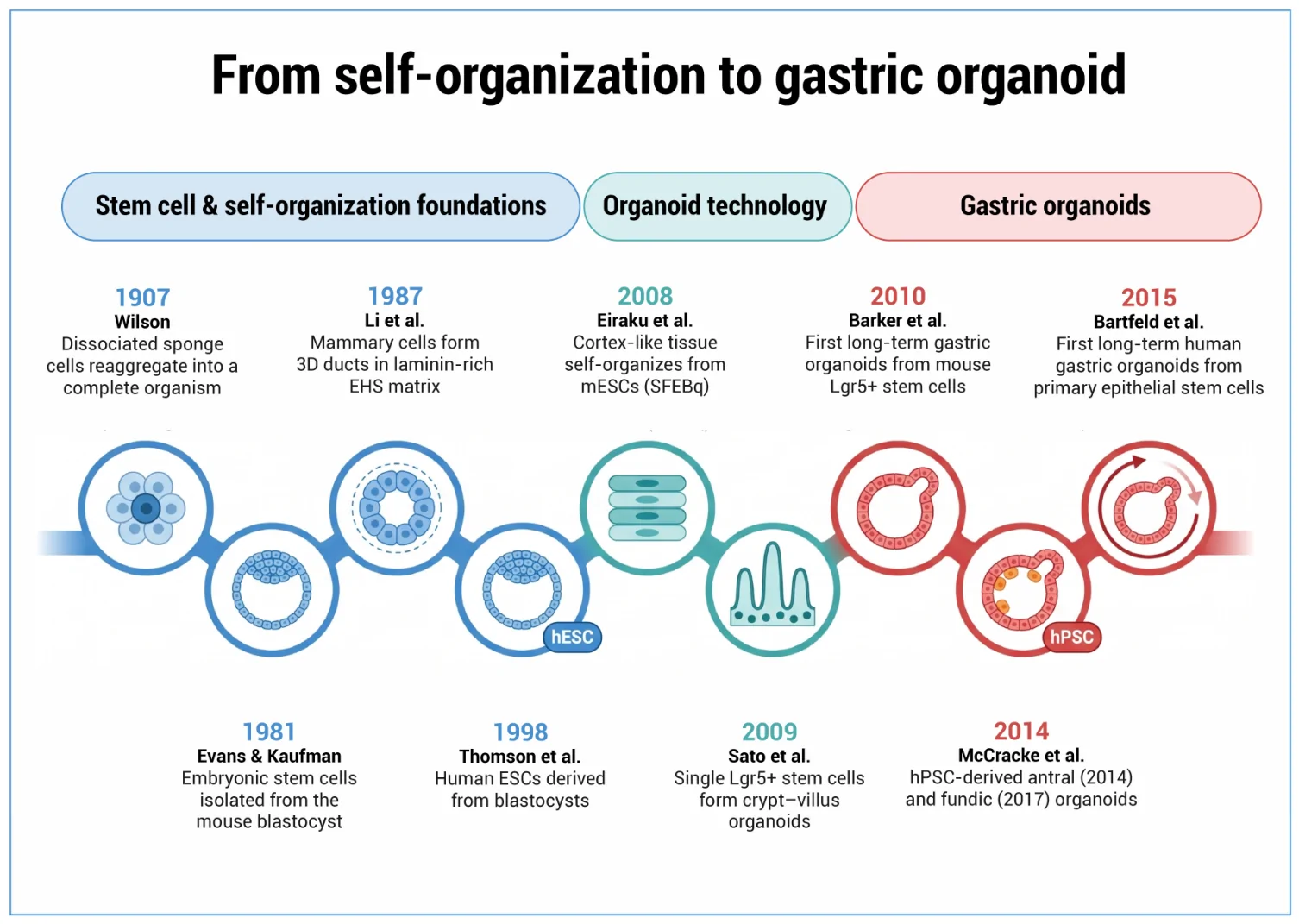
Timeline of key milestones leading to the development of gastric organoids. Foundational studies on cellular self-organization and stem cell biology (blue) include the demonstration of self-organization in dissociated sponge cells (Wilson, 1907) [27], the isolation of mouse (Evans and Kaufman, 1981) [28] and human (Thomson et al., 1998) [29] embryonic stem cells, and the culture of mammary epithelial cells in laminin-rich extracellular matrix (Li et al., 1987) [30]. Advances in organoid technology (teal) include the self-organization of cortex-like tissue from mouse embryonic stem cells (Eiraku et al., 2008) [31] and the generation of intestinal organoids from single Lgr5^+^ stem cells (Sato et al., 2009) [32]. Gastric organoid milestones (red) include the first long-term gastric organoids from mouse Lgr5^+^ stem cells (Barker et al., 2010) [19], human pluripotent stem cell-derived antral and fundic organoids (McCracken et al., 2014, 2017) [17,18], and long-term cultures of primary human gastric epithelial stem cells (Bartfeld et al., 2015) [6]. The timeline is not drawn to scale, and icons are schematic. ESC, embryonic stem cell; hESC, human ESC; hPSC, human pluripotent stem cell; EHS, Engelbreth–Holm–Swarm; SFEBq, serum-free floating culture of embryoid body-like aggregates with quick reaggregation. (Created in BioRender. Zahra Enteshari Najafabadi, Ireneusz Ryszkiel, Amir Sadeghi. (2026). https://BioRender.com/d2p411h).

The concept of self-organization in biology, which is the basis for organoid technology, was first proposed in 1907. At that time, Henry van Peters Wilson created a complete organism in the laboratory using isolated sponge cells, providing an important conceptual basis for the subsequent development of organoid culture [27]. The key milestones leading to gastric organoids are summarized in Figure 1.

In 1981, embryonic stem cells (ESCs) were first isolated from the inner cell mass of mouse blastocysts, marking a major leap forward in the field [28]. Nearly two decades later, in 1998, Thomson et al. established human ESCs from blastocysts [29]. At the same time, extensive research efforts were underway to improve the conditions for in vitro culture. In the early 1980s, attention was focused on 3D culture systems that more closely resembled the in vivo environment [33]. In 1987, Li and colleagues showed that mammary epithelial cells could form 3D structures of ducts and lumens in a laminin-rich extracellular matrix [30].

The first major breakthrough in the field of organoids was achieved by Eiraku et al. in 2008. They showed that mESCs in serum-free 3D culture conditions, in a method called SFEBq, were able to self-organize into structures that closely resembled the cerebral cortex without the need for patterning cues [31]. A year later, in 2009, Sato et al. demonstrated the ability of *Lgr5*^+^ intestinal stem cells to generate intestinal organoids. Remarkably, these organoids were able to differentiate into crypt-villus structures without the need for a mesenchymal niche [32]. This work accelerated the application of organoid technology to other tissues, including the stomach.

The first gastric organoid was successfully generated in 2010. This organoid derived from mouse antrum glands was made in a medium containing FGF10 and the hormone gastrin. In this study, the expression of chief cell- and mucus neck cell-specific markers (PGC and MUC6, respectively) was detected. In contrast, reducing WNT concentration induced the generation of mucosal and endocrine gland lineages. However, no parietal cells were observed [19]. Gastric organoids were subsequently derived from *Troy*^+^ chief cells of the murine gastric corpus, which act as reserve stem cells in vivo and can differentiate toward mucous neck and pit cell lineages in culture [25].

Building on this initial success in creating mouse gastric organoids, researchers have attempted to tailor protocols for developing human organoids. One of the most notable advances came in 2014 when McCracken and colleagues developed the first gastric organoid differentiation protocol from human PSCs. Antral organoids contained *LGR5*-expressing cells, surface mucous cells, antral gland cells, and enteroendocrine cells [17]. The group also optimized the protocol to generate fundic organoids and used a MAPK/ERK kinase (MEK) pathway inhibitor to generate parietal cells [18].

In 2015, Bartfeld et al. established a novel, long-term, and stable 3D organoid culture system for both primary, untransformed human gastric epithelial cells and human gastric cancer cells [6]. These stem cells could be expanded indefinitely and directed to differentiate into various gastric lineages. This model provides a platform for studying gastric biology and host–pathogen interaction [6]. More recent developments have expanded gastric organoids toward multicellular and more physiologically relevant models, an evolution discussed in subsequent sections (Table 2A,B).

## 4. Sources and Protocols for Gastric Organoid Formation

As discussed in the introductory section, organoids have emerged as a transformative tool in biological research, providing a useful platform for studying complex tissue morphogenesis and modeling human diseases [45,46]. These systems are generated using two major cell sources: PSCs and ASCs. They enable the establishment of 3D cultures. Importantly, the choice of cell source profoundly impacts the characteristics, differentiation potential, and applications of the resulting organoids [4].

### 4.1. Generation of Gastric Organoids from Pluripotent Stem Cells

ESCs, derived from the inner cell mass of the blastocyst, can be maintained in an undifferentiated state under specific culture conditions [29,47]. In addition to ESCs, iPSCs can be obtained by reprogramming somatic cells through the ectopic expression of Yamanaka factors (Oct4, Sox2, Klf4, and c-Myc) [48]. These cells possess the ability to self-renew and differentiate into a variety of cell types, including those of the gastric epithelium [49].

While 2D culture systems enable the differentiation of PSCs into various lineages, including gastric epithelial cells, they fail to recapitulate the organ morphology and cellular diversity. 3D culture techniques, such as organoid culture, have been used to overcome these limitations. Notably, PSCs can be differentiated into gastric organoids that primarily contain epithelial cells and may also include a mesenchymal component [17,46].

The generation of gastric organoids from PSCs follows a step-by-step protocol mimicking key stages of embryonic stomach development. These organoids contain different types of differentiated and functional gastric epithelial cells surrounded by undifferentiated mesenchymal cells (Table 2A).

**Figure 2:**
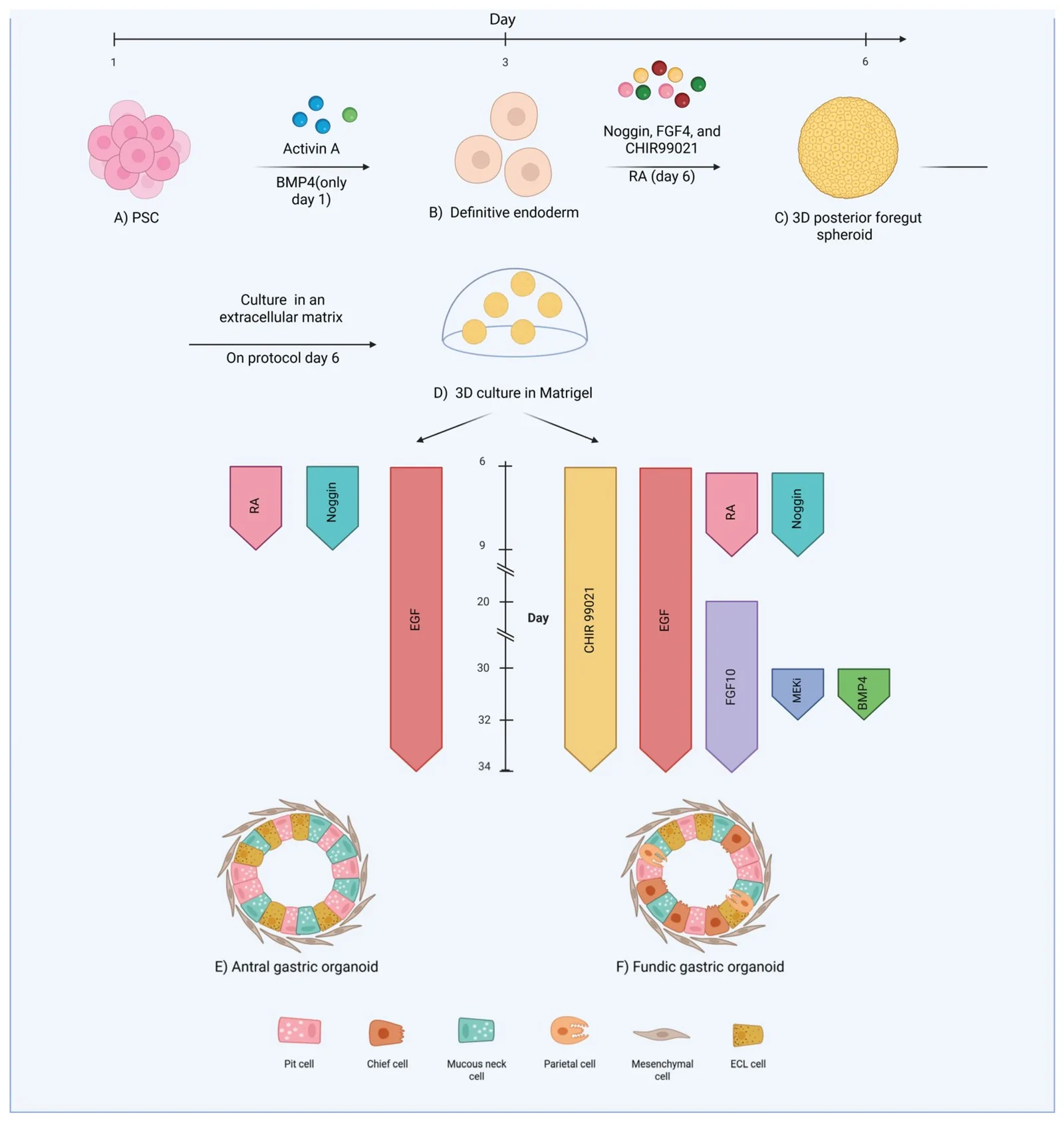
Schematic protocol for generating gastric organoids from pluripotent stem cells. (**A**) Pluripotent stem cells (PSCs) are differentiated into definitive endoderm during days 0–3 using Activin A and BMP4 (BMP4 is applied only on day 1). (**B**) Definitive endoderm is patterned into posterior foregut between days 3–6 through treatment with FGF4, CHIR99021, and retinoic acid (RA), while BMP signaling is inhibited by Noggin to promote foregut fate. (**C**) By day 6, posterior foregut cells self-organize into three-dimensional spheroids. (**D**) On day 6, spheroids are embedded in an extracellular matrix such as Matrigel. (**E**) Antral gastric organoids are induced in EGF-containing medium with RA, with Noggin treatment limited to days 6–9, giving rise to mucous neck, pit, and endocrine cells. (**F**) In the continuous presence of EGF and CHIR99021, posterior foregut spheroids are directed toward a fundic gastric fate. While Noggin and RA are present in the medium from days 6 to 9, FGF10 is applied from days 20 to the end of the protocol, while BMP4 and the MEK inhibitor PD0325901 are transiently applied from days 30 to 32 to promote parietal cell differentiation, resulting in fundic gastric organoids containing pit, neck, chief, parietal, and endocrine cell types. (Created in BioRender. Zahra Enteshari Najafabadi, Ireneusz Ryszkiel, Amir Sadeghi. (2026). https://BioRender.com/d2p411h).

Initially, PSCs are directed toward the DE lineage by exposing them to a member of the TGF-β-family, activin A, which induces Nodal signaling. Subsequently, DE cells undergo posterior foregut specification through treatment with RA, FGF4, WNT, or a GSK3β inhibitor such as CHIR99021 [15,17,50]. At this stage, the addition of Noggin is essential to inhibit BMP4 signaling and promote a foregut fate. These posterior foregut cells are then embedded in an extracellular matrix, such as Matrigel, to facilitate 3D spheroid formation [17]. To generate antral organoids, the spheroids are treated with RA, Noggin and epidermal growth factor (EGF), leading to the differentiation of specific gastric epithelial cell types, including mucous neck cells and pit cells, as well as enteroendocrine cells, particularly gastrin-producing G cells that are antrum-specific. This process typically spans approximately 34 days (Figure 2) [17].

However, Wnt signaling should be triggered to induce differentiation toward the gastric corpus lineage. Therefore, the culture medium is supplemented with CHIR99021, EGF, and FGF10. To specifically promote parietal cell development, BMP4 and a MEK inhibitor (PD0325901) are incorporated into the culture conditions [18]. Upon differentiation, corpus organoids recapitulate the cellular diversity of the gastric corpus, comprising pit cells, mucous neck cells, endocrine cells, chief cells, and acid-secreting parietal cells, which are essential for gastric physiology [35].

A similar strategy is employed for the generation of gastric organoids from mouse PSCs. Initially, PSCs are aggregated into embryoid bodies and exposed to sonic hedgehog (SHH), the Wnt pathway inhibitor Dickkopf-related protein 1 (DKK1), and Noggin [22]. Activation of SHH signaling, in conjunction with WNT inhibition, induces the formation of primitive tubular structures reminiscent of the early embryonic stomach. These spheroids are subsequently embedded within an extracellular matrix scaffold and cultured in the presence of FGF10, Noggin, WNT, and R-spondin, leading to the emergence of corpus gastric glands within approximately 60 days [22].

In this approach, gastric organoids are derived from PSCs, which can be maintained and extensively expanded in vitro, are readily available for laboratory use, and can be generated from patient-derived cells, although their practical scalability is constrained by lengthy multistep differentiation, specialized expertise, cost, and line-to-line variability. PSCs can also be used to generate different types of organoids. In PSC-derived organoids, mesenchymal cells are derived from a small non-endodermal population and increase in number within spheroids embedded in Matrigel. Furthermore, these organoids can serve as a useful model for studying *H. pylori* infection due to their ability to recapitulate key features of the gastric epithelial cells, particularly acid-secreting parietal cells.

### 4.2. Establishing Gastric Organoids Using ASCs

Organoids can also be derived from ASCs that originate from the same tissue. Gastric tissue stem cells are primarily found in the isthmus and base of the fundic and antral glands of the stomach [3,4,19,25]. These cells possess the ability to differentiate into various gastric epithelial cell types and, under appropriate conditions, can be cultured to form 3D organoids [6] (Table 2B).

**Figure 3:**
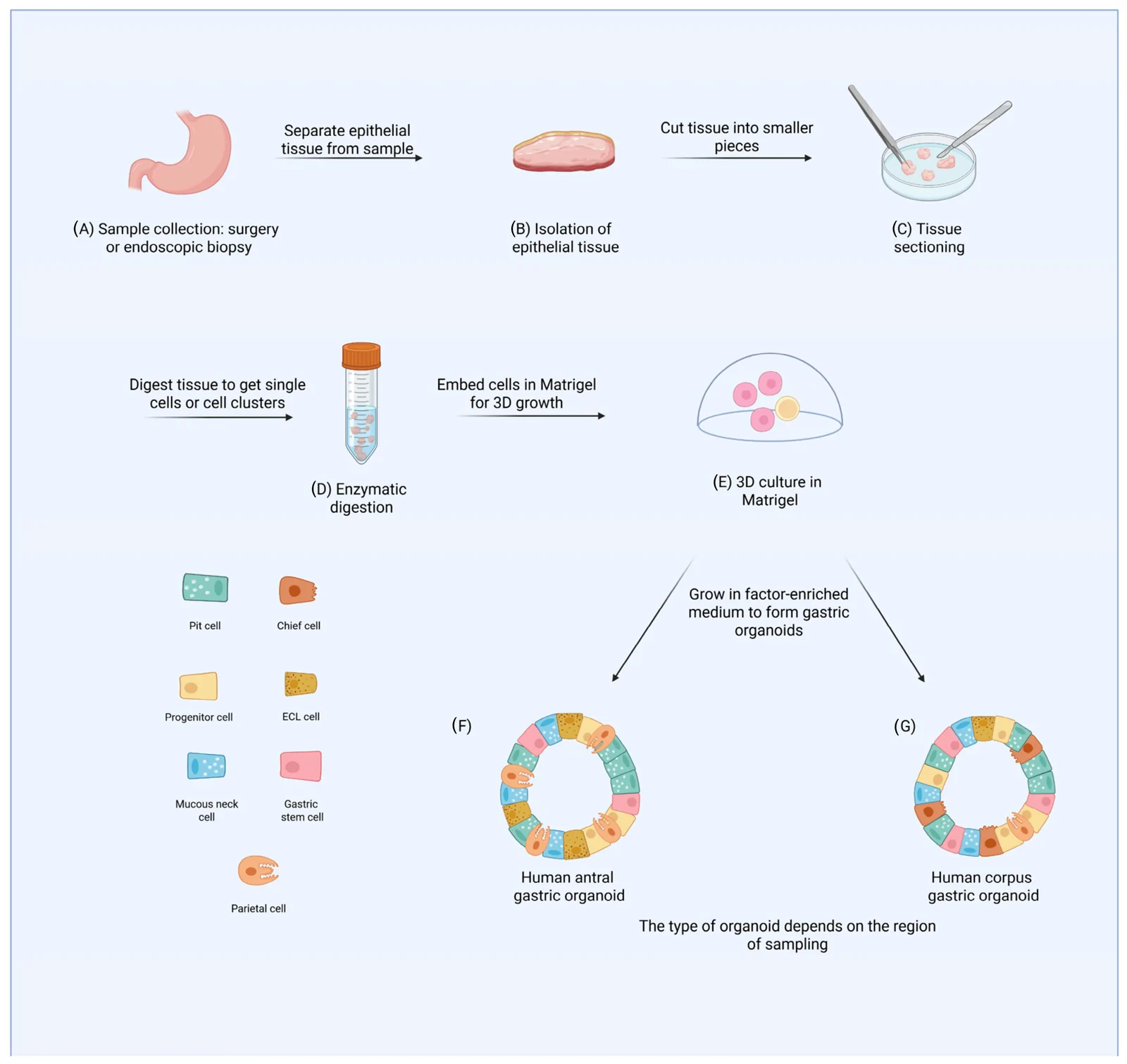
Schematic steps for generating gastric organoids from ASCs. (**A**) First, gastric tissue is sampled, usually done by endoscopy or surgery. (**B**) The epithelial tissue is isolated and (**C**) divided into smaller pieces. (**D**) These pieces are then subjected to enzymatic digestion (such as collagenase) to obtain single cells or cell aggregates containing gastric stem cells (especially Lgr5^+^ cells). (**E**) These extracted cells and glands are seeded in a matrix such as Matrigel that mimics the in vivo environment and provides 3D growth conditions. Finally, using a culture medium enriched with growth factors, including FGF10, EGF, Gastrin, WNT3A, R-spondin1, and Noggin, gastric organoids are formed, which contain functional gastric epithelial cell types. For human culture, TGF-β inhibitors are also used to enhance long-term survival and favorable differentiation. These organoids can differentiate into (**F**) human antral organoids or (**G**) human corpus organoids, depending on the source of sampling. ASC-derived organoids are usually formed in 7–14 days, are genetically stable, and resemble mature gastric tissue closely. (Created in BioRender. Zahra Enteshari Najafabadi, Ireneusz Ryszkiel, Amir Sadeghi. (2026). https://BioRender.com/d2p411h).

The first step in generating gastric organoids from ASCs involves isolating gastric tissue, typically through endoscopy or surgery. The tissue is then dissected into smaller fragments and digested, often using collagenase, to release gastric glands containing stem cells (Figure 3) [6]. These glands are enriched in *Lgr5*^+^ stem cells, which serve as progenitors for organoid culture. The extracted glands and stem cells are then combined with an extracellular matrix such as Matrigel, which mimics aspects of the in vivo environment and supports cell growth and differentiation [6].

Gastric organoid culture requires a specialized culture medium containing various growth factors (Figure 3). For mouse-derived organoids, factors such as FGF10, EGF, and gastrin are used to stimulate growth and differentiation. Furthermore, Wnt signaling is one of the key factors in this process. Research has shown that the factors WNT3A and R-spondin1, which activate the Wnt/β-catenin signaling pathway, are essential for the formation and expansion of organoids [19,51]. These factors help maintain the stem cell niche and self-renewal of cells in organoids [6]. R-spondin, a potent WNT agonist, enhances Wnt signaling through the LGR5/ring finger protein 43 (RNF43) axis [52]. By forming a complex with LGR4/5 and inhibiting the activity of RNF43 and ZNRF3, R-spondin causes cells to express sufficient levels of the WNT receptor (Frizzled) on their plasma membrane [53]. This mechanism leads to stable and continuous activation of Wnt signaling and helps to maintain self-renewal and differentiation of stem cells in organoids [23,54]. In addition, Noggin, a BMP antagonist, should be added to the culture medium to prevent stem cell differentiation. For human gastric organoids, in addition to these factors, TGF-β inhibitors are required to provide optimal conditions for cell growth and differentiation and increase the longevity of organoids [6].

A major advantage of ASC-derived organoids is their rapid formation, typically within 7 to 14 days. These organoids are predominantly rich in gastric epithelial stem cells and, depending on the growth factors in the culture medium, can differentiate into a variety of mucosal, endocrine, and other gastric cell types [4,6,19]. Furthermore, ASC-derived organoids are genetically highly stable, and when provided with the appropriate cellular niche, they possess the ability to self-renew and to be maintained in culture over the long term [6].

In contrast, PSC-derived organoids are generated through a stepwise differentiation protocol that guides PSCs through endodermal induction, foregut formation, and ultimately differentiation into the gastric epithelium [17,18,46]. While this approach allows the generation of a wider range of gastric cell types, including mesenchymal, endocrine, and even parietal cells, the formation process is much longer, typically taking 30 to 60 days. Additionally, PSC-derived organoids are more prone to genetic instability over time and require more stringent culture conditions [46]. In general, ASC-derived organoids may be particularly useful for gastrointestinal disease modeling and tissue repair studies because they can be generated relatively rapidly, maintain genetic stability, and more closely recapitulate mature gastric tissue than PSC-derived organoids [6,46].

The choice of the appropriate method depends on the research goal and the required complexity of the biological model.

## 5. Advances

Since the initial introduction of gastric organoids, extensive efforts have been made to develop 3D culture systems that better recapitulate the complex structure and function of gastric tissue. Early organoid models, particularly those derived from ASCs, were limited to epithelium-only cultures and lacked mesenchymal and other stromal components. These models also faced challenges such as immature cellular differentiation and a trade-off between long-term maintenance and full maturation. The success of organoid culture systems relies on the precise supplementation of niche-specific growth factors that recapitulate the native stem cell microenvironment. Furthermore, gastric cancer organoids present challenges, including contamination by normal epithelial cells and intra-tumoral heterogeneity, limiting their utility in research and therapeutic applications [4,39]. Table 3 summarizes cell culture systems, detailing their advantages, limitations, and applications.

### 5.1. Air–Liquid Interface

Air–liquid interface (ALI) culture system is a specialized culture method that exposes the apical surface of epithelial cells to air while maintaining the basal surface in contact with culture medium [56]. This approach was used by Ootani et al. to generate intestinal organoids [59]. In the ALI system, cells are placed in a 3D matrix, such as collagen. This collagen is placed on a dish with a porous bottom membrane. The membrane is subsequently added to a second plate. This arrangement allows for exposure to air on one side and the liquid medium on the other, creating the ALI. The ALI enhances the oxygenation of the cells, which is essential for the long-term culture of epithelial cells [37,56].

In this context, an innovative study employing the ALI system successfully established a 3D culture of mouse gastric epithelial cells under conditions that closely mimic the native stem cell niche. In this approach, neonatal mouse gastric cells are embedded in a collagen gel composed of Cell matrix type I-A, Ham’s F-12 medium, and a regeneration buffer, and cultured at an air–liquid interface within a porous membrane insert. This configuration facilitates the formation of “gastrospheres”—spherical structures comprising a polarized inner epithelial monolayer surrounded by an outer layer of myofibroblasts. Remarkably, this system supports epithelial cell viability for over 80 days, in contrast to prior methods that were limited to approximately one week. The gastrospheres exhibit key physiological features, including MUC5AC expression, apical microvilli, secretory granules, and a high turnover rate [37].

In addition, the ALI system facilitates the differentiation of gastric epithelial cells into mucosal surface cells. Furthermore, the ALI and the early development of myofibroblasts are essential for the growth and maturation of gastric epithelial cells. In the absence of the ALI system, cell survival is reduced, and the formation of cell spheres is sporadic and limited [59].

This model allows the study of epithelial dynamics, differentiation, and cellular interactions in a quasi-in vivo environment, without the need for specialized reagents, and holds strong potential for gastric disease modeling and drug screening [60].

### 5.2. Extracellular Matrix-Based Culture

Matrigel, derived from the Engelbreth–Holm–Swarm (EHS) mouse sarcoma, is an extracellular matrix (ECM) extract rich in basement membrane proteins, widely used as a scaffold in cell culture and in vivo studies. Containing key components such as laminin, type IV collagen, entactin, and heparan sulfate proteoglycans, this substrate provides a biomimetic extracellular environment that facilitates the growth and differentiation of cells in three dimensions [66].

One of the main advantages of Matrigel is its ability to promote cell differentiation. Studies have shown that many cell types such as epithelial cells of the pancreas, salivary glands, and mammary glands form structures similar to natural tissues when cultured on Matrigel [66]. Matrigel, by forming a 3D substrate at 37 °C, enables self-organization of cells and is a useful model for studying biological processes in organoids. Studies have also shown that Matrigel plays an important role in angiogenesis, such that endothelial cells form capillary structures with lumens in the presence of this scaffold, allowing the study of cell–matrix interactions in a quasi-physiological environment [57].

Given these properties, Matrigel has been widely employed in gastric organoid cultures as a key component of the culture system. Following the isolation of gastric glands or single cells from tissue, these cells are embedded in Matrigel, which provides a 3D extracellular matrix-like environment essential for organoid formation. Matrigel supports spatial organization, apical-basal polarity, and the development of a central lumen, features that are critical for the proper differentiation of gastric stem cells and the in vitro recapitulation of gastric epithelium. Moreover, by preserving cellular phenotypes and enabling long-term expansion, Matrigel establishes a useful platform for studying both physiological processes and disease mechanisms, including *H. pylori* infection [6].

In addition to Matrigel-based 3D culture systems that support the growth and differentiation of gastric stem cells, conversion to 2D culture can facilitate direct cellular access and enable high-resolution experimental manipulation. In this method, growing organoids are first mechanically disrupted by pipetting and then enzymatically dissociated to obtain single cells. These cells are cultured in a specialized 2D culture medium and in the absence of 3D-inducing stimuli on collagen-coated wells—commonly type I or IV collagen. The collagen coating plays a key role in cell attachment and maintaining epithelial polarity. Due to its structural similarity to the natural basement membrane, the collagen substrate promotes effective cell adhesion, epithelial island formation, and ultimately the formation of homogeneous and polarized monolayers in a short period of time [5].

2D cultures, while preserving the characteristics of differentiated cells, have several advantages, especially in modeling *H. pylori* infection, including easy access to the cell surface for imaging and infection studies, reduced morphological variation, and ease of performing high-throughput experiments. In addition, this system is able to recreate pathological features associated with infection, such as the “hummingbird” phenotype, cytokine secretion, and CagA phosphorylation [5].

Despite its many advantages, Matrigel has limitations that need to be addressed in related research. A primary limitation of Matrigel is its tumor-derived origin, which leads to batch-to-batch variability and lack of precise composition. Additionally, because Matrigel is derived from murine Engelbreth–Holm–Swarm sarcoma tissues, undefined batch-to-batch variations in growth factors and basement membrane constituents limit its reproducibility for standardized disease modeling and translational studies [58]. Another limitation of Matrigel is the lack of precise control over the microenvironment; although this scaffold provides a 3D environment, it lacks the ability to accurately reproduce the stiffness and other mechanical signals of different tissues. Finally, another important limitation is the constraint on long-term expansion, as static Matrigel domes lack functional vasculature, ultimately restricting passive diffusion of oxygen and nutrients as organoids grow beyond diffusion limits [4,67].

Despite these limitations, Matrigel remains one of the most widely used scaffolds in organoid research because it provides a suitable environment for cell growth, differentiation, and organization. Alternative matrices differ mainly in how well their composition is defined: natural matrices such as collagen I, which has been used in ALI cultures of gastric cells [37,59]; hydrogels derived from decellularized tissue; engineered recombinant ECM-protein hydrogels; and fully synthetic hydrogels [68] (Table 4). Most of the evidence for these alternatives comes from intestinal organoids, and gastric-specific data are limited.

Synthetic hydrogels, such as PEG-based systems, are fully defined, with independently tunable stiffness, degradability, and biochemical motifs [70,74,75]. While modular PEG matrices have primarily been used in intestinal organoids to define divergent mechanical requirements for stem-cell self-renewal versus differentiation [74,75], gastric applications have similarly emerged, as shown by the successful maintenance of human gastric organoids and dendritic cell recruitment within synthetic VitroGel^®^ systems [73]. Nevertheless, such gastric implementations currently serve as preliminary feasibility demonstrations within short-term microfluidic or co-culture platforms rather than comprehensive validation for long-term serial passaging, multi-lineage differentiation, or mature parietal cell acid secretion [73]. Consequently, defining fully synthetic or recombinant formulations capable of supporting the complete phenotypic spectrum of the gastric epithelium across extended culture remains an ongoing objective [68,73].

In dome-embedded 3D culture, hydrogels derived from decellularized tissue are the main non-Matrigel alternative for which gastric data are available, whereas collagen I has been used predominantly in ALI formats [37,59]. Hydrogels derived from decellularized extracellular matrix retain native biochemical signatures and have been directly evaluated against Matrigel in gastric organoids [70,71,72]. A hydrogel derived from decellularized porcine small intestine supported pediatric gastric organoid derivation from primary tissue without Matrigel. This porcine hydrogel was produced with a pipeline designed to be compatible with good manufacturing practice (GMP) [70]; however, certified clinical-grade GMP manufacture of decellularized matrices for gastric organoids has not yet been demonstrated. A decellularized porcine stomach ECM hydrogel supported gastric organoids with viability and morphology comparable to those observed in Matrigel [72]. Human stomach-derived ECM hydrogels supplemented with 25% Matrigel were associated with reduced expression of genes related to cellular stress and inflammation compared with Matrigel alone [71]. Nonetheless, direct comparisons indicate that parietal cell differentiation remains incomplete in decellularized matrices relative to mature tissue, and batch-to-batch variability across tissue harvests necessitates rigorous lot testing [70,72].

In summary, a critical distinction must be made between preliminary feasibility studies, which demonstrate short-term viability or specialized microfluidic compatibility, and comprehensive validation frameworks required for translational applications. While direct pairwise comparisons have demonstrated the feasibility of tissue-derived hydrogels against Matrigel, broad and standardized head-to-head benchmarking across multiple synthetic and natural matrices under long-term culture conditions is still lacking. Standardized evaluation of defined hydrogels during serial passaging, cryopreservation, and functional differentiation is therefore essential before non-animal matrices can reliably replace Matrigel in clinical workflows.

### 5.3. Co-Culture Systems

ASC-derived gastric organoids are predominantly epithelial and generally do not reproducibly retain the stromal and immune compartments of the original tissue [4,6,19]. PSC-derived gastric organoids can contain mesenchymal populations, although their abundance and composition depend on the differentiation protocol; additional mesenchymal cells have been incorporated into some models to improve tissue complexity [16]. Mesenchymal cells provide WNT ligands and other paracrine niche signals [76,77], and epithelial–mesenchymal co-culture promotes differentiation in mouse fundic organoids [78]. ALI culture has also maintained gastric epithelium and myofibroblasts in neonatal mouse tissue for more than 80 days [37]. However, these findings do not establish long-term preservation of the immune microenvironment in human gastric tumors. Evidence from intestinal organoids further demonstrates the potential of incorporating additional lineages, including enteric neurons [79]; gastric neuromuscular models are discussed in Section 5.5. Likewise, vascular components remain less explored in gastric organoids than in intestinal models, where hPSC-derived organoids contain a transient endothelial population that can be maintained under modified culture conditions [80].

For gastric cancer, the key question is which immune and stromal populations each model contains, how long they persist, and whether their interactions with tumor epithelium remain functionally representative (Table 5). Gastric-specific evidence includes models supplemented with autologous T cells, peripheral blood mononuclear cells (PBMCs), or cancer-associated fibroblasts, whereas evidence for sustained endogenous immune-cell retention in ALI cultures and for perfused tumor–immune microfluidic systems remain limited in the studies reviewed. In six peritoneal-metastasis organoid lines, cytotoxicity induced by allogeneic PBMCs was not explained by HLA matching alone [81]. However, alloreactivity could not be excluded, and tumor-antigen-specific recognition was not established. Autologous tumor-reactive or appropriately primed T cells, together with controls for antigen specificity and functional antigen presentation, provide a more informative framework for evaluating tumor-directed responses [82,83]. Myeloid representation remains a particular limitation: myeloid-derived suppressor cells were incorporated in one autologous gastric cancer model [82], whereas dendritic cells were investigated in non-malignant gastric organoids [73,84]. Among the studies identified, no gastric tumor model combined autologous lymphocytes, myeloid populations, and organized stromal architecture with validation against clinical immunotherapy outcomes. These limitations constrain the use of gastric PDOs for predicting immunotherapy responses.

Overall, the principal challenge is not simply to increase cellular diversity, but to preserve the appropriate cellular composition, antigen-specific immune function, and spatial organization of the gastric tumor microenvironment. Future models should therefore be evaluated against these complementary criteria rather than cellular inclusion alone.

### 5.4. Organoid-on-a-Chip

One of the advanced platforms for organoid culture is the organoid-on-a-chip system [62]. Research in this field has led to the development of two prominent gastric organoid-on-a-chip models: 3D gastric organoid (3DGO)-on-a-chip and 2D monolayer gastric organoid (2DMGO)-on-a-chip. Both models have demonstrated utility in recapitulating human physiology and disease modeling; however, they differ significantly in terms of architecture, functionality, and cellular heterogeneity, making each more suitable for specific applications [61,62].

3D models incorporate microfluidics to create dynamic conditions such as nutrient flow and mechanical stimulation. In general, the architecture of these chips allows researchers to manipulate fluid flow, pressure, and biochemical gradients to mimic blood circulation or nutrient delivery in vivo [64].

In this regard, the organoid-on-a-chip platform was introduced to develop more physiologically relevant in vitro models for studying human stomach function. Hong’s group designed a microfluidic system to recapitulate aspects of gastric luminal dynamics and mechanically induced motility-like movement. The main body of the chip was fabricated from PDMS (polydimethylsiloxane), a biocompatible and transparent material with oxygen permeability, which is ideal for constructing such platforms due to its ease of handling and the absence of complex instrumentation. At the center of the chip, a chamber was designed to embed a stomach organoid (derived from the antrum) in a Matrigel substrate, flanked by two side chambers that house the culture medium. To establish luminal flow, two borosilicate glass micropipettes (with an orifice diameter of approximately 50 μm) were positioned opposite each other on either side of the organoid. These micropipettes were connected to a peristaltic pump via tubing. Prior to injection, the flow path was filled with a fluorescent dextran-FITC solution, which was then slowly infused into the organoid lumen via the pump. This system not only enables real-time visualization of material flow, but also provides a platform for the long-term delivery of nutrients or pharmacological agents into the gastric lumen [34].

The designed platform is capable of inducing rhythmic stretching and contraction of the organoid, mimicking certain mechanical features associated with gastric motility. This functionality is enabled by a three-roller peristaltic pump, which, during each rotation cycle, generates subtle pressure fluctuations in the tubing and, consequently, volumetric changes within the organoid lumen. These fluctuations manifest as sequential expansions and contractions of the organoid. Thus, this platform provides a physiologically relevant microfluidic model of the human stomach in vitro, integrating 3D organoid growth, luminal perfusion, and motility-like movements. This system represents a promising tool for preclinical studies, gastrointestinal disease modeling, and oral drug screening. Moreover, since it can be combined with patient-derived organoids, it also offers a platform for potential precision-medicine application [34].

In contrast, 2DMGO-on-a-chip models offer significant advantages in accessibility, stability, and experimental versatility by rapidly forming uniform and homogeneous layers of epithelial cells. In these models, the apical surface is directly exposed to the culture medium, allowing easy access to both sides of the epithelium and enabling the selective examination of surface responses, an important feature for studies such as innate immune responses. These 2D models can also be cultured under ALI conditions, inducing morphological changes that resemble native tissue architecture and promote mucus production. By providing a controllable environment for co-culture with different cell types, including fibroblasts and endothelial cells, these systems facilitate detailed investigation of cellular interactions and molecular pathways involved in both normal and pathological functions of the gastric mucosa. Jantaree et al. developed a prototype multilayer design in which the gastric epithelium was positioned on an upper porous membrane, while fibroblasts and endothelial cells were located in the underlying compartments. This structural arrangement enables targeted investigation of intercellular interactions, responses to pathogens, and layer-specific secretions using independent microfluidic channels [61].

In a recent effort, Hofer et al. developed a human gastric organoid-on-a-chip system designed to study the physiological and pathological functions of the gastric epithelium. This model, like previous systems, is fabricated from a PDMS chip; however, its hydrogel substrate enables bidirectional experimental access to both the apical and basal surfaces of the epithelium. Gastric epithelial cells derived from organoid cultures are seeded onto this hydrogel substrate and gradually form a uniform and homogeneous monolayer [65]. In this system, the culture medium can be independently modulated on the apical and basal sides. To mimic physiological conditions, the apical compartment contains medium with reduced levels of growth factors, whereas the basal side is supplied with medium enriched in these factors to simulate the underlying mesenchymal environment. This design maintains epithelial stability and integrity for up to 13 days without compromising its native geometry. Most importantly, the platform can establish physiologically relevant apical acidic conditions, which are essential for accurately modeling gastric function and make this system particularly well-suited for studying the establishment and chronic colonization of *H. pylori* in the gastric epithelium [65].

### 5.5. Neuromuscular Integration and Gastric Motility

Previous sections described systems that primarily recapitulate gastric epithelial functions, including differentiation, secretion, and response to infection; however, gastric motility is not recapitulated in these models. This motility results from the coordinated interplay between smooth muscle cells (SMCs), interstitial cells of Cajal (ICC), and the enteric nervous system (ENS). Existing approaches have only partially addressed this gap: the incorporation of enteric neurons into intestinal organoids (Section 5.3) demonstrated neuron-dependent contractile activity in non-gastric tissue, whereas organoid-on-a-chip platforms (Section 5.4) induce stretching and contraction movements via external mechanical stimulation rather than through an intrinsic motor circuitry. Early hPSC-derived gastric organoids [17,35] lacked organized smooth muscle, ICC, and an ENS; this gap drove the development of subsequent multicellular approaches [16]. The following section examines advancements that specifically target this gap within gastric tissue (summarized in Table 6).

In hiPSC-derived gastric organoids, it has been shown that epithelium-secreted SHH and TGF-β1 acted on the subepithelial mesenchyme to induce its differentiation into SMCs, which formed a continuous muscle band (muscularis mucosae) beneath the epithelium during long-term 3D culture; however, contractile activity of this layer was not reported [93]. In three-germ-layer gastric organoids, in which hPSC-derived splanchnic mesenchyme and enteric neural crest cells (ENCCs) generated separately from the foregut endoderm were combined with it and followed by in vivo engraftment, distinct muscularis mucosae and muscularis externa layers formed around the epithelium [16]. In mouse ESC-derived intestinal-like organoids (non-gastric), SMCs likewise organized into parallel layers and contracted spontaneously [95]. Physical confinement of gastric tissue growth similarly yielded a muscle layer capable of generating rhythmic contractile force [94]. Collectively, these studies illustrate progression from the mere structural presence of smooth muscle to organized layers and measurable contractile activity. However, the presence of SMCs, or even spontaneous contractions, does not equate to recapitulation of gastric motor function; the rhythmic nature of these contractions does not, in itself, establish the mechanisms generating or regulating this rhythmic activity.

Beyond the generation of contractile muscle layers, the next step was reconstructing the electrophysiological mechanism responsible for rhythmic activity. In three-germ-layer human gastric organoids, c-KIT^+^ ICCs formed endogenously within a region analogous to the muscularis externa and were associated with spontaneous phasic contractions [16]. More direct functional evidence has been obtained from non-human models. In mouse ESC-derived intestinal-like organoids, c-KIT^+^ multipolar cells formed a dense interconnected network within the muscle layer [95]. ANO1 co-expression with c-KIT, a characteristic feature of the pacemaker lineage, was reported in bioengineered intestinal muscularis [97]. However, the expression of these markers alone does not establish pacemaker function. Direct recording of the electrophysiological activity in the mouse model demonstrated spontaneous slow waves and pacemaker potentials that persisted in the presence of nifedipine, indicating that pacemaker activity originates from the ICCs independently of smooth muscle excitation [95]. In bioengineered intestinal muscularis complexes, blocking ANO1 with niflumic acid abolished periodic contractions, consistent with a dependence of rhythmic motor activity on ANO1-mediated Cl^−^ currents [97]. In contractile organoids derived from the embryonic chick hindgut, calcium transients in c-KIT^+^ ICCs preceded those in SMCs, and this temporal relationship was abolished by gap junction inhibition, supporting ICC-to-smooth muscle signal transmission [96]. In three-germ-layer human gastric organoids, however, ICCs have been identified by marker expression and localization [16], and direct recording of electrical slow waves or other mature electrophysiological activity has not yet been reported [16,95,96].

The integration of the ENS with muscle tissue represents a step beyond the mere presence of neurons in gastric organoids; existing evidence indicates that ENCCs can establish a functional neuromuscular unit. In three-germ-layer gastric organoids, ENCCs migrated through the mesenchyme and organized into ganglionic structures and nerve bundles resembling myenteric and submucosal plexuses, adjacent to SMCs and c-KIT^+^ ICCs. Their addition transformed the irregular contractions of non-innervated tissue into regular, periodic contractile waves. In tissue strips from in vivo-engrafted three-germ-layer gastric organoids, low-intensity electrical field stimulation (EFS), insufficient to depolarize smooth muscle directly, elicited contractile waves that were sensitive to TTX, a voltage-gated Na^+^ channel blocker [16]. In confinement-based gastric tissue, an ENS emerged de novo without exogenous ENCCs, and EFS-induced contractions in the resulting tissues were significantly reduced by TTX [94]. In human intestinal organoids containing an ENS (Section 5.3), the nicotinic agonist DMPP induced smooth muscle relaxation that was blocked by TTX and by the nitric oxide synthase inhibitor L-NAME [79]. In the same gastric tissue strips, EFS-evoked contractile waves were likewise modulated by L-NAME and attenuated by atropine, a muscarinic receptor antagonist, consistent with the concurrent involvement of an inhibitory nitrergic pathway (nNOS^+^ neurons) and an excitatory cholinergic pathway (ChAT^+^ neurons) in the regulation of contraction [16]. However, in all these studies, neuronal activity was evoked by an external stimulus, namely EFS or DMPP [16,79,94,98], and it remains unclear whether these tissues can generate neurally coordinated motor responses to mechanical or chemical mucosal stimuli without artificial electrical or pharmacological stimulation.

To our knowledge, despite these advances, no gastric organoid model has yet combined the epithelium, smooth muscle, ICCs, and ENS with functional characterization of all four components (Table 6). The closest gastric models, three-germ-layer gastric organoids and confinement-based gastric tissues, contain cells expressing markers of all four components [16,94], yet their functional characterization is uneven: muscle contraction and neural modulation have been examined using EFS combined with TTX and cholinergic/nitrergic pharmacology, whereas ICCs have been identified on the basis of c-KIT expression and spatial localization alone [16,94]. Direct slow-wave recordings, ANO1 blockade, and calcium imaging of ICCs have not been reported in any gastric organoid model, whereas direct evidence for ICC pacemaker activity and ICC–SMC coupling comes only from non-gastric models [95,96,97]. The contribution of ICCs to the rhythmic contractions of gastric tissues therefore remains inferred rather than established. Physiological gastric motility patterns, including directional peristalsis, a regional pacemaker hierarchy, and receptive relaxation, have likewise not been reported in any gastric organoid model; the only motility-like mechanical deformation described is externally imposed by pump-driven flow in an organoid-on-a-chip system [34]. The experimental approaches established in non-gastric models, including intracellular microelectrode recordings, pharmacological blockade of ANO1 and gap junctions, and calcium imaging of ICC–smooth muscle activity, could in principle be applied to gastric organoids to investigate gastric slow-wave generation, ion-channel function, and neuromuscular activity. Finally, maturation of the muscularis externa and neural networks in human gastric models depends on several weeks of in vivo engraftment, and none of these models incorporates extrinsic (vagal or sympathetic) innervation [16,94]. Neurohormonal regulation of gastric motility has likewise not been addressed in gastric organoid models to our knowledge, representing an additional gap beyond the neuromuscular components discussed above.

These neuromuscular constraints are particularly relevant to gastroparesis. Full-thickness gastric biopsies from patients with diabetic and idiopathic gastroparesis show loss of ICCs, injury to the remaining ICCs, an abnormal immune infiltrate containing macrophages, and reduced nerve fibers [99]. In diabetic gastroparesis, ICC loss was associated with delayed gastric emptying [100], and altered expression of ANO1 splice variants has been reported [101]. To our knowledge, no gastric organoid model of gastroparesis has been described, and because ICC identity in gastric organoids currently rests on c-KIT expression and localization rather than demonstrated pacemaker function, existing models are not yet suited to investigating these abnormalities.

Similar constraints apply to other non-obstructive motor disorders, such as functional dyspepsia (FD). A major pathophysiological subset of patients with FD exhibits impaired gastric accommodation—characterized by defective postprandial relaxation of the proximal fundus [102,103]. Physiologically, gastric accommodation is mediated by a vagovagal neuro-humoral reflex that culminates in intrinsic nitrergic (nNOS^+^) enteric neuronal relaxation of fundic smooth muscle [104]. Although engineered three-germ-layer gastric organoids develop nNOS^+^ neurons capable of L-NAME-sensitive relaxation [16], current models do not fully recapitulate the extrinsic neural inputs and physiological distension required to study gastric accommodation, nor do they reproduce the coordinated reflex response or permit comprehensive assessment of dynamic gastric wall compliance. Consequently, existing organoid systems primarily enable the investigation of individual components of nitrergic relaxation rather than the integrated vagovagal reflex underlying gastric accommodation or its impairment in FD.

In contrast, gastrointestinal stromal tumors (GISTs) highlight the limitations of epithelial-centric models in capturing mesenchymal oncogenesis. GISTs are thought to arise from the ICC lineage and are commonly driven by mutually exclusive activating mutations in KIT [105] or PDGFRA [106]. The ETS-family transcription factor ETV1 acts as a lineage-survival factor that cooperates with oncogenic KIT, while MAPK signaling contributes to ETV1 protein stabilization [107,108]. Standard epithelial-focused patient-derived organoid protocols are not designed to preserve the mesenchymal and ICC-lineage components required to model GIST. To our knowledge, no gastric organoid model of GIST or functional dyspepsia has been reported; the only GIST-derived organoid culture we identified was described in a single case report of a patient with liver metastasis, in which the organoids were sensitive to sunitinib but tolerant to imatinib [109]. Although multilineage organoids can generate endogenous ICC-like cells [16,94], their suitability for modeling GIST requires further validation, including assessment of ETV1 expression, ANO1-associated pacemaker function, and the trophic support needed for long-term ICC maintenance. A precedent for neuromuscular disease modeling exists in human intestinal organoids containing an ENS, in which *PHOX2B*-mutant enteric neural crest cells were used to model Hirschsprung disease [79]. Thus, developing disease-relevant organoid models of GIST and neuromuscular disorders will require improved preservation of mesenchymal cell populations, validation of ICC identity and function, and functional integration of the relevant neural and smooth-muscle components.

Combining multicellular gastric tissues with organoid-on-a-chip platforms is a logical next step but remains prospective. Current gastric chips either impose rhythmic deformation externally through pump-driven pressure changes [34] or provide perfusion and bilateral apical/basal access to epithelial cultures [61,65], whereas three-germ-layer and confinement-based gastric tissues show spontaneous contractile activity and neurally modulated responses but depend on in vivo engraftment for maturation [16,94]. In principle, integrating these approaches would allow intrinsic neuromuscular activity to be examined under controlled luminal flow, acidity, and distension, and would permit the recording and imaging approaches established in non-gastric models to be applied [95,96,97]. To our knowledge, however, no gastric system combining a multicellular neuromuscular tissue with a microfluidic platform has been reported, and its functional validation remains to be established.

### 5.6. Artificial Intelligence in Organoid Culture and Analysis

In a research laboratory working with organoids, artificial intelligence can support image analysis tasks such as segmentation and growth monitoring [110,111]. Its role extends further when organoid phenotypes are linked to molecular data and drug responses, thereby aiding in the characterization of disease heterogeneity. Studies have illustrated this at each level: bright-field images of patient-derived lung cancer organoids were used to predict gene expression [112]; a NanoString panel combined with supervised classifiers assigned molecular subtypes in gastric cancer organoids paired with their parent tissue [113]; and PharmaFormer, pretrained on cell-line data and fine-tuned on patient-derived organoids, produced predictions associated with survival in TCGA cohorts [114]. These findings, however, come from small numbers of organoid lines or samples and require experimental validation [112,113], and whether image-based expression prediction and drug-response modeling extend to gastric organoids remains to be tested. Clinical application in gastric cancer requires larger datasets, standardization, and independent validation [115].

## 6. Application

### 6.1. Disease Modeling

**Figure 4:**
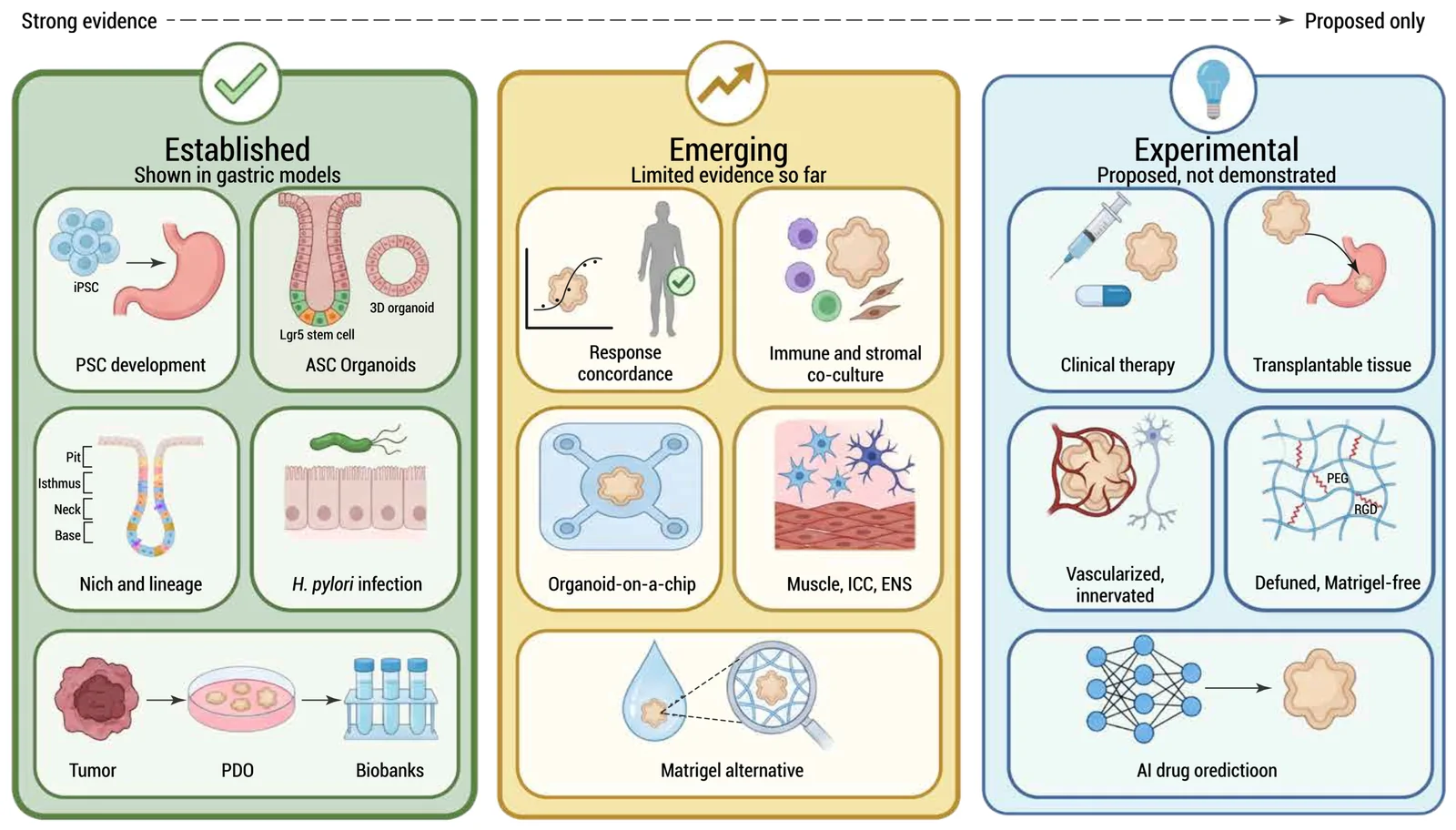
Translational maturity and evidence levels in gastric organoid research. Overview of gastric organoid technologies categorized from established capabilities to experimental horizons: (**Left**) established platforms with consistent gastric-specific validation, comprising PSC and ASC differentiation, gastric stem cell niche modeling, *H. pylori* pathogenesis, and tumor-derived PDO biobanks. (**Center**) Emerging approaches characterized by preliminary or evolving evidence, including retrospective patient-response concordance, stromal/immune co-cultures, stomach-on-a-chip technologies, neuromuscular integration (smooth muscle, ICCs, ENS), and non-Matrigel ECM hydrogels. (**Right**) Experimental frontiers currently unproven or proposed in human gastric models, encompassing organoid-guided clinical treatment decisions, transplantable tissue replacement, complete vascularized/innervated stomach models, defined synthetic matrices, and deep learning-based drug prediction. (Created in BioRender. Zahra Enteshari Najafabadi, Ireneusz Ryszkiel, Amir Sadeghi. (2026). https://BioRender.com/cdnpemc).

Organoids can serve as models for investigating the pathophysiology of various diseases, encompassing infectious diseases, cancer, and genetic disorders. Through the use of organoid culture, researchers can gain insights into disease progression and identify potential therapeutic targets [6] (Figure 4).

#### 6.1.1. Gastric Organoids to Study *Helicobacter pylori* Pathogenesis and Drug Screening

*H. pylori* is a spiral, Gram-negative bacterium that infects approximately 50% of the world’s population, and its prevalence varies greatly in different regions. *H. pylori* survives in the acidic environment through urease activity and complex regulatory pathways [116]. The BabA2 adhesin of *Helicobacter pylori* has been associated with duodenal ulcer and distal gastric adenocarcinoma in clinical isolates [117]. More importantly, *H. pylori* plays a role in the development of gastric adenocarcinoma [84]. East Asian strains carrying cagA are more strongly associated with gastric cancer than Western strains [116].

Various animal models have been employed to study *H. pylori* infection; however, each has specific limitations in recapitulating the pathogenesis of this bacterium in humans [118]. The Mongolian gerbil is widely recognized as one of the most common and efficient animal models, as it supports persistent colonization by cag-positive strains and develops chronic active gastritis, gastric ulcers, intestinal metaplasia, and, in a number of studies, adenocarcinoma [119,120,121]. Specifically, infection with the type I strain B128 or its gerbil-adapted derivative 7.13 induces corpus-dominant atrophic gastritis, hypochlorhydria, and hypergastrinemia, and these alterations depend on a functional cag type IV secretion system (T4SS) [122,123]. This is in contrast to wild-type mice, in which immune-driven recombination in cagY frequently reduces T4SS function during chronic infection. Although cagY-dependent loss of T4SS function has also been reported in gerbils, some gerbil-adapted strains have retained T4SS function [124]. Nonetheless, because gerbils are outbred, dissecting host-related factors is challenging, and genetic manipulation and systematic analysis of host responses remain limited [125].

The house musk shrew (*Suncus murinus*) is another small mammal of interest. *Helicobacter suncus* was isolated from the stomach of shrews with chronic gastritis [126], and its pathogenicity has been evaluated in this host [127]. Gastric mucosa and physiology of this animal resemble those of humans and dogs [128]. However, evidence for the susceptibility of *S. murinus* to *H. pylori* itself remains limited, and its suitability as a model requires further investigation.

In contrast, wild-type mouse strains such as C57BL/6 [129] and BALB/c [130] typically develop only mild disease and fail to reproduce peptic ulcers or gastric cancer. Structural differences in the murine stomach, a distinct microbiota, and the inability of many *H. pylori* strains to trigger strong inflammatory responses further limit their relevance. Transgenic mice deficient in regulatory cytokines or T regulatory cells can develop severe gastritis, but their immunological complexity makes interpretation difficult [118]. Non-human primates are costly and time-intensive, and it remains unclear whether macaques are natural hosts [131]. Furthermore, guinea pig studies largely predate modern metagenomic approaches, leaving this model insufficiently characterized [118]. Finally, gastric cancer cell lines offer a controlled environment but lack the cellular and architectural complexity of the intact gastric epithelium and may mislead interpretations of early gastric carcinogenesis [132].

**Figure 5:**
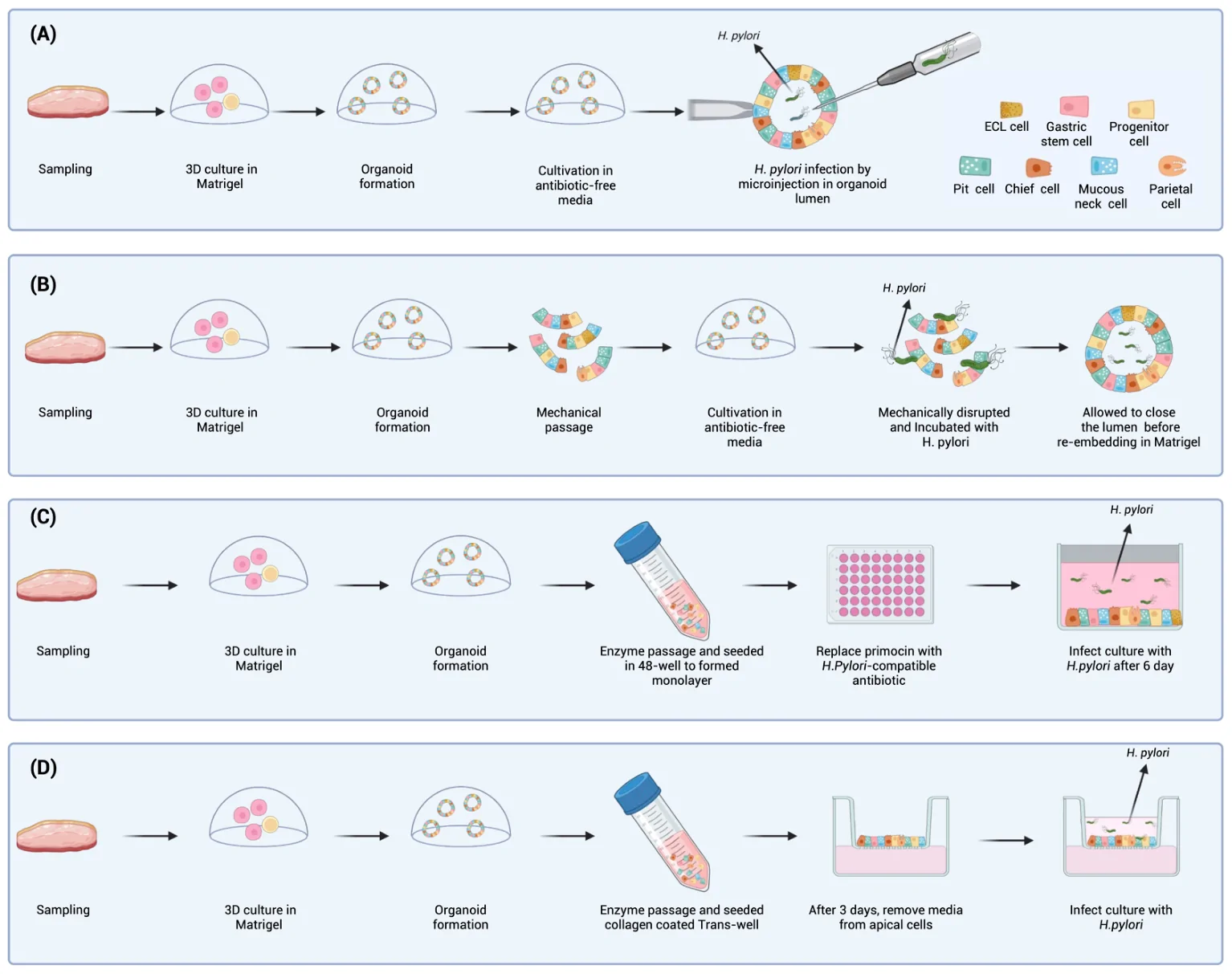
Different methods for establishing an *H. pylori* infection model. (**A**) In this method, after organoid establishment, infection with *H. pylori* is induced by microinjection. (**B**) In this method, organoids are formed, and mechanical passage is performed using a Pasteur pipette to expose the apical surface of the organoid. Then, organoid pieces are exposed to bacteria, and infection is confirmed after 4 h of incubation. (**C**) In this method, after organoid establishment, enzymatic passage is performed, and cells are cultured in 48-well plates to form a monolayer. After a few days, when the cell monolayer reaches 100% confluency, bacteria are added to the culture medium and incubated for 6 h to induce infection. (**D**) This procedure is mainly used to establish the ALI system; a monolayer is grown on a Transwell rather than a conventional well plate. (Created in BioRender. Zahra Enteshari Najafabadi, Ireneusz Ryszkiel, Amir Sadeghi. (2026). https://BioRender.com/bqtd1xl).

Organoids have emerged as advanced and valuable models in studies of *H. pylori* infection, offering distinct advantages over traditional animal models and 2D cell lines. They preserve cellular diversity, including mucous, chief, parietal, and enteroendocrine cells, and maintain a 3D structure reminiscent of the gastric epithelium [132,133]. Infection of organoids has been induced by microinjection, mechanical fragmentation, conversion of 3D cultures into 2D monolayers, or infection of ALI culture [5,65,134,135] (Figure 5) (Table 7).

Acute gastritis caused by *H. pylori* is characterized by overexpression of IL-8, a key player in the central pathway of innate immunity that causes infiltration of multinucleated neutrophils and promotes inflammation [63]. Studies by Bartfeld et al. have demonstrated that organoids derived from glandular cells differ from those derived from the pit lineage. In these models, organoids were purposefully differentiated into two types, “pit-type” and “gland-type”, through specific environmental conditions. Following microinjection of *H. pylori* into the lumen of the organoids, a markedly higher expression of inflammatory genes, particularly IL-8, was observed in gland-type organoids compared to pit-type counterparts. This indicates that glandular cells play a central role in initiating the primary immune response to *H. pylori* infection. Complementary microarray analyses revealed that among the genes upregulated more than twofold upon infection, the human chorionic gonadotropin beta subunit gene (*CGB*), which has previously been associated with gastric cancer, exhibited the highest level of induction. In this model, activation of the NF-κB pathway was confirmed as a key axis of the epithelial inflammatory response, evidenced by nuclear translocation of the p65 subunit in infected organoids, a phenomenon which was absent in neighboring uninfected organoids. The recorded immune response notably included IL-8 secretion in a dose-dependent manner, which was triggered even by aflagellated bacteria. In contrast, LPS and CpG failed to induce any response, suggesting a distinctive sensitivity profile of these organoids to classical immune stimuli [6]. Another study introduced a primary culture system of human gastric epithelial cells derived from healthy tissue, enabling a more accurate reconstruction of early epithelial responses to *H. pylori* infection. In this system, gastric glands were isolated from human biopsy samples and cultured in Matrigel supplemented with defined growth factors. This led to the formation of 3D spheroids with quasi-unlimited proliferative potential, which could be differentiated into gastric organoids by the withdrawal of WNT3A and R-spondin1. A key innovation of this model is the ability to convert these 3D structures into polarized, 2D monolayers of epithelial cells, which are particularly well suited for direct analysis of *H. pylori* infection [5] (Figure 5).

The organoid model has also enabled the study of the response of parietal cells. Loss of SHH expression in parietal cells leads to phenotypic changes in gastric epithelium [138]. Studies have shown that SHH expression increases in acid-secreting parietal cells after *H. pylori* microinjection into fundic organoids derived from Shh::GFP mice. These findings highlight the importance of organoid models in understanding cellular signaling responses to *H. pylori* infection [134].

In the context of studying the mechanisms of pathogenesis and host responses to *H. pylori* infection, organoids have enabled the elucidation of key aspects such as inflammation, tissue damage, disruption of homeostasis, bacterial adhesion to the epithelial surface, intracellular effects of virulence factors (such as CagA), activation of innate immunity, and stimulation of epithelial cell proliferation, processes that could not previously be accurately investigated using conventional models. In this regard, studies have shown that after entering host cells, CagA is phosphorylated by tyrosine kinases such as Src and Abl and interacts with the cMET receptor, leading to increased cell proliferation and morphological changes such as cell elongation [139]. The presence of CagA in infected organoids results in the activation of the cMET/CD44/HGF pathways, which play a key role in host cell proliferation [140]. Research has also shown that the formation of the CagA–ASPP2 complex is required for altering cellular organization and facilitating bacterial colonization. Furthermore, this study suggested that kinase inhibitors, particularly those targeting the EGFR/PI3K/AKT signaling pathway, could act as potential agents to disrupt this interaction, thereby reducing the ability of *H. pylori* to colonize the stomach [141]. Studies in organoids derived from CD44-deficient mice have shown that cell proliferation is notably impaired in the absence of the CagA–ASPP2 complex [140]. Researchers also demonstrated that *H. pylori* preferentially binds to highly differentiated pit cells, which are characterized by high levels of GKN1, GKN2, and PSCA [44].

Despite its advantages, the gerbil model is limited by the outbred background of the animals and the scarcity of immunological reagents compared with mice [136]. Organoids derived from susceptible species could therefore complement in vivo infection studies. Establishing gastric organoids from gerbils or shrews would allow genetic manipulation and mechanistic dissection of host–pathogen interactions under controlled in vitro conditions, with simpler tissue sampling and a smaller number of animals than in vivo experiments, while preserving the epithelial architecture. Moreover, such models would enable comparison of species-specific epithelial responses with those of human- and mouse-derived organoids [5,6,134]. However, research in this area is still at an early stage. To our knowledge, the only organoid system reported from *S. murinus* is an intestinal one, which was generated to study motilin secretion and could be passaged and maintained for six months or longer [142], and no published reports of gastric organoids derived from either gerbils or *S. murinus* could be identified. Therefore, the development and characterization of gastric organoids from these susceptible hosts remains an unexplored but promising direction.

Beyond *H. pylori*, Epstein–Barr virus (EBV) is another pathogen implicated in gastric carcinogenesis, and EBV-associated gastric cancer is recognized as a distinct molecular subtype [143] that accounts for approximately 10% of gastric cancers [144]. The role of EBV co-infection in *H. pylori*-related cancers has remained unclear owing to the lack of efficient ex vivo models [144]. Microinjection of EBV and *H. pylori* into patient-derived normal gastric organoids showed that co-infection altered organoid morphology, increased proliferation, and upregulated *TFF1*, *VIL1*, and *LGR5* [144]. Electron microscopy also suggested increased intracellular localization of *H. pylori* after co-infection, although the virus-like particles observed require further confirmation [144]. Nevertheless, this evidence comes from an epithelium-only model followed for up to 96 h [144], and whether EBV and *H. pylori* act synergistically in the presence of immune and stromal components remains to be established.

More recently, gastric organoid models have been used to evaluate potential therapeutic strategies against *H. pylori*. A recent study assessed potential interventions targeting components of the NF-κB pathway [145]. Overall, organoids have become useful biological models for investigating *H. pylori* pathogenicity, including its interaction with other pathogens such as EBV, and evaluating potential therapeutic strategies by more closely replicating the gastric environment, especially compared to 2D cell cultures. Figure 5 illustrates various approaches for establishing an *H. pylori* infection model.

#### 6.1.2. Gastric Organoids and COVID-19

Although SARS-CoV-2, the causative agent of COVID-19, primarily targets the respiratory tract, clinical evidence also indicates gastrointestinal symptoms such as diarrhea and vomiting [146]. In this context, human gastric organoid models have provided a relevant system to directly investigate the effects of the virus on gastrointestinal epithelial tissues.

In one study, 3D human gastric organoids were employed to explore the route of viral entry and the host epithelial response to SARS-CoV-2 infection. The findings demonstrated that the gastrointestinal epithelium is susceptible to infection via the apical membrane, with infection inducing an epithelial inflammatory response. These observations support the use of this model for studying gastrointestinal viral infection and offer a platform for screening targeted antiviral therapeutics [147].

However, evidence from region-specific gastric organoid models indicates that susceptibility to SARS-CoV-2 may vary according to gastric maturity and tissue state. Mature gastric epithelium exhibits resistance to SARS-CoV-2 infection, primarily attributed to the absence of ACE2 expression, the principal receptor for viral entry. Importantly, ectopic expression of ACE2 alone rendered the organoid-derived cells susceptible to SARS-CoV-2, supporting productive viral replication [148]. Consistent with this developmental distinction, gastric organoids derived from pediatric and late-fetal tissue showed greater susceptibility to SARS-CoV-2 infection and higher levels of viral replication. These findings highlight a potential role for the stomach in gastrointestinal transmission of SARS-CoV-2, particularly in children [43].

Taken together, these studies indicate the mature gastric epithelium is relatively resistant to SARS-CoV-2 in the absence of ACE2, whereas immature gastric tissues, particularly pediatric and late-fetal tissues, may exhibit greater susceptibility to infection via the apical surface. Beyond these specific findings, such studies illustrate how organoids can be used to define tissue tropism and entry mechanisms of emerging pathogens and to screen candidate antivirals during an ongoing outbreak [149,150]. This is particularly relevant given that coronaviruses have caused three major outbreaks since 2002 (SARS-CoV, MERS-CoV, and SARS-CoV-2) [151], suggesting that such platforms may be valuable for preparedness against future emerging coronaviruses. Nevertheless, the ASC-derived epithelial organoids used in these studies lack immune and stromal components, and modeling luminal viral exposure requires specialized approaches such as apical-out (reverse polarity) organoids [43] or organoid-derived monolayers [148].

#### 6.1.3. Gastric Organoids to Model Gastric Cancer and Evaluate Drug Response

Gastric cancer is one of the most common malignant tumors of the digestive system and ranks fifth among the most common cancers in the world in terms of incidence. According to GLOBOCAN 2022, more than 960,000 new cases and approximately 660,000 deaths were reported globally [152]. This persistent burden underscores the need for experimental models that better capture the biological complexity of the disease.

Intestinal-type gastric cancer is thought to develop through a multistep sequence, known as the Correa cascade, in which normal gastric epithelium progresses through chronic gastritis and atrophy to intestinal metaplasia (IM), dysplasia, and finally adenocarcinoma [153]. Organoids derived from different stages of this sequence provide an epithelial platform for studying individual transitions. In mouse models, organoid lines established from the stomachs of *Mist1-Kras* (G12D) mice at metaplastic and dysplastic stages showed that *Kras* activation controls progression to dysplasia. Dysplastic organoids responded to MEK inhibition, although the surviving cells retained cellular heterogeneity [154]. In humans, a cohort of 28 IM organoid lines, compared with 42 normal gastric organoid lines, displayed a spectrum from hybrid gastric/intestinal to advanced intestinal differentiation, suggested a possible origin of IM from STMN1-expressing cycling isthmus stem cells, and revealed an increased mutation burden and frequent chromosome 20 gain [155].

Studies applying risk factors to gastric organoids have so far been conducted mainly in mouse-derived systems. Organoids isolated from *H. felis*-infected mice showed increased expression of intestine-related genes, such as *Isx*, *Muc4*, and *Vil1*, and decreased expression of gastric genes, including pepsinogen C and *Gif* [156]. Deoxycholic acid, the main bile acid of duodenogastric reflux, activated STAT3 signaling and its downstream intestinal marker KLF5 in mouse gastric organoids [157], whereas treatment with the carcinogen N-methyl-N′-nitro-N-nitrosoguanidine (MNNG) induced *Cdx2* and *Muc2* expression and reduced *Atp4b* and *Muc6* in mouse corpus organoids, with metaplastic cells arising from MIST1^+^ chief cells [158]. Notably, these different stimuli converge on a limited number of regulatory hubs. Forced CDX2 expression reprogrammed mouse stomach organoids toward a hybrid stomach–intestinal state resembling clinical metaplasia, while HNF4A induced weaker intestinalization by activating a *Cdx2* shadow enhancer [159]. Likewise, *CDX2* overexpression in human iPSC-derived gastric organoids induced intestinal phenotypes, although incompletely [160]. Conversely, loss of Cdx2 converted intestinal stem cells into gastric-type stem cells in organoid culture [14], supporting a role for *CDX2* as a determinant of epithelial identity. RAS–MEK signaling [154,158] and the STAT3–KLF5 axis [157] represent additional nodes in this pathway.

Evidence for reversibility in organoid models is limited and restricted to marker-level readouts. In patient-derived gastric intestinal metaplasia organoids from three patients, taurine increased *MUC5AC* and decreased *CDX2*, *MUC2*, and *TFF3* expression [161], and eradication of *H. felis* altered the stem/progenitor marker profile of organoids derived from infected mice [156]. Whether such changes reflect a stable restoration of gastric lineage identity remains to be determined. Moreover, most of these models are epithelium-only, derived from mouse tissue or from small numbers of human lines, and therefore do not capture the contribution of the immune and stromal microenvironment to metaplasia progression.

In addition to the sporadic intestinal-type pathway described above, organoids have been used to model hereditary diffuse gastric cancer (HDGC), a syndrome caused by germline variants in *CDH1*, which encodes E-cadherin, and associated with a high lifetime risk of diffuse gastric cancer and, in women, lobular breast cancer [162]. In gastric organoids derived from *Cd44*-*Cre*/*Cdh1^loxP^*^/loxP^ mice, single-cell RNA sequencing showed that *Cdh1* inactivation shifted cells along a squamous differentiation trajectory, and cytokeratin 7 was identified as a marker of early neoplastic lesions in *CDH1* mutation carriers [163]. In human gastric organoids, CRISPR/Cas9-mediated E-cadherin knockout led to progressive loss of the single-layered epithelial architecture and detachment of individual cells; mechanistically, E-cadherin deficiency disrupted the spindle orientation machinery, so that daughter cells were positioned outside the epithelial layer, mimicking early diffuse-type lesions [164]. To investigate malignant progression and niche factor autonomy, patient-derived and CRISPR-engineered gastric organoids demonstrated that compound *CDH1* and *TP53* mutations drive tumorigenesis by conferring independence from exogenous R-spondin [39]. In mouse organoids, *Cdh1* knockout combined with Kras^G12D^ and Trp53 knockout accelerated tumorigenesis with features of immune evasion compared with Kras^G12D^/Trp53 knockout alone [165].

Nevertheless, these models have limitations. Most rely on complete *CDH1* knockout, which represents total loss of function and may not reflect the heterogeneity of patient-specific germline variants, particularly missense variants of uncertain significance [166]. In addition, in the studies identified here, organoid work relevant to the breast manifestation of the syndrome was limited to mouse mammary organoids [167]. Among the studies identified in our review, no model derived from non-neoplastic gastric tissue of germline *CDH1* carriers was identified.

Gastric cancer treatment faces several challenges, such as marked intratumoral heterogeneity and limitations of currently available biomarkers for predicting response to therapy [168]. Targeted therapies are further limited by primary and acquired resistance; for example, resistance to trastuzumab in HER2-positive disease remains an important challenge [169]. Similarly, PD-L1 expression assessed by the combined positive score (CPS) is an imperfect predictor of immunotherapy benefit, owing to debated cutoffs, spatial heterogeneity, and interobserver variability [170]. Preclinical models that faithfully capture tumor genomics, phenotype, and drug response while preserving the tumor microenvironment (TME) remain limited, and the dynamic complexity of the TME, despite its central role in treatment efficacy, is difficult to reproduce in vitro [171].

To better understand the extensive molecular heterogeneity of gastric cancer, various classifications have been proposed. The Lauren classification system divides gastric cancer into three types based on histological characteristics: intestinal, diffuse, and mixed [172]. Another classification system, The Cancer Genome Atlas (TCGA), divides gastric adenocarcinoma into four molecular subtypes, including EBV-positive subtype (with frequent mutations in *PIK3CA* and silencing of the *CDKN2A* gene), microsatellite instability (MSI) subtype (with a hypermutated phenotype and silencing of *MLH1* and *MSH6*), genomically stable (GS) subtype (with diffuse histology and mutations in *CDH1* and *RHOA*), and chromosomal instability (CIN) subtype (with aneuploidy, frequent mutations in *TP53*, and activation of the RTK–RAS pathway) [143]. In addition to TCGA, the Asian Cancer Research Group (ACRG) has also proposed a different classification system for gastric cancer, which includes MSI, *MSS*/*TP53*-, *MSS*/*TP53*^+^, and MSS/epithelial–mesenchymal transition (EMT) subtypes [173]. MSI-high and EBV-positive subtypes appear more responsive to immune checkpoint inhibitors, with the strongest clinical evidence for MSI-high tumors [174]. Immune checkpoint blockade has also entered the perioperative management of resectable gastric cancer, as the phase 3 MATTERHORN trial evaluated the addition of the anti-PD-L1 antibody durvalumab to FLOT chemotherapy in resectable gastric or gastroesophageal junction adenocarcinoma [175]. Durvalumab plus FLOT significantly improved event-free survival compared with placebo plus FLOT (hazard ratio 0.71) [175]. The final analysis subsequently showed a significant overall survival benefit (hazard ratio 0.78; 95% CI 0.63–0.96) [176]. Because epithelium-only organoids cannot capture such immune-mediated effects, these findings underscore the need for models that retain the tumor immune microenvironment [171].

The use of cancer cell lines, 2D culture and animal models was valuable in early research and investigation of the basic mechanisms of cancer development. However, each of these approaches has limitations that may reduce their ability to capture patient-specific therapeutic responses. Animal models also face logistical, ethical, and economic constraints, while humanized models may not fully recapitulate the complexity of human immune interactions within the tumor microenvironment (TME). Similarly, cell lines have limited reliability for predicting patient response owing to their limited resemblance to the primary tumor, restricted molecular heterogeneity, transcriptomic drift, and absence of key TME components, including immune cells. These limitations underscore the need to develop more physiologically relevant models, such as patient-derived organoids, for translational research and preclinical drug screening [171,177,178]. Normal gastric organoids recapitulate key features of the native gastric epithelium, including lineage-specific differentiation and stem cell self-renewal [6,19,179]. Unlike conventional cell lines, which have limited representation of tumor heterogeneity and the TME, patient-derived organoids (PDOs) have been reported to largely retain the morphological, histopathological, genomic and transcriptomic profiles of the primary tumor. In this review, PDOs refer to organoids established from patient tumor tissue (also termed tumor-derived or gastric cancer organoids), whereas organoids established from non-malignant tissue are referred to as normal or ASC-derived gastric organoids. However, standard PDOs are epithelial cultures and lack the stromal and immune components of the tumor microenvironment, which must be added through co-culture or advanced platforms. These models can represent distinct molecular subtypes such as MSI, EBV-positive, GS, and CIN, and exhibit diverse ex vivo responses to chemotherapeutic agents and targeted therapies [41].

Beyond their resemblance to patient tumor tissue, organoids are experimental platforms for assessing patient-specific therapeutic responses and investigating intrinsic and acquired drug resistance [38,40,42,180]. However, whether PDO-based drug testing can reliably guide treatment selection and improve patient outcomes in routine clinical practice remains to be established. Moreover, advanced culture systems such as the ALI or organoid-on-a-chip platforms have been developed to incorporate immune components into organoid-based models. These systems may enable investigation of immune–tumor interactions and exploratory testing of immunotherapies, including PD-1 blockade; however, their validity for predicting immunotherapy response in gastric cancer remains to be established.

In this regard, Seidlitz et al. established a CIN model by combining *Kras*^G12D^ and *Tp53*^R172H^ mutant alleles in mouse gastric organoids, which recapitulated key histopathological features of this subtype, including a thickened and irregular epithelium, nuclear accumulation of *Tp53*, and enriched Erk1/2 phosphorylation. Additionally, a GS model with Wnt activation (GS-WNT) was generated through targeted deletion of Apc and *Cdh1*, resulting in a grape-like morphology that reflects the loss of E-cadherin–mediated cell–cell adhesion typical of diffuse-type gastric cancer. These mouse-derived models provide experimental platforms for evaluating targeted therapies. For instance, inhibition of the β-catenin–TCF complex led to the downregulation of Wnt target gene expression [41].

PDOs are valuable 3D models established from tissue specimens obtained via surgical resection or endoscopic biopsies. Following tissue acquisition, samples undergo enzymatic dissociation, and the resulting cells are embedded within an extracellular matrix and cultured in a defined medium. Several research groups [38,39,40,41,42] have successfully generated multiple lines of both tumor-derived and normal gastric organoids that largely retain the genetic, morphological, and histopathological features of the original tumors. These models provide experimental platforms for evaluating responses to chemotherapy and targeted agents and for investigating tumor-specific therapeutic vulnerabilities which may inform future potential precision-medicine applications, pending prospective validation. Among these, the study by Nanki and colleagues introduced a distinctive approach to organoid generation by employing a niche factor-based positive selection strategy to prevent the overgrowth of normal organoids and actively enrich for malignant clones. This methodology involves the use of selective pressures, such as Nutlin-3 to isolate *TP53*-mutant organoids, removal of ROCK inhibitor to enrich for RHO-pathway–dysregulated tumors, and withdrawal of EGF/FGF10 to select for organoids with ligand-independent RTK pathway activation. These refined conditions improved the efficiency of gastric cancer organoid derivation and yielded models with molecular profiles closely resembling the parental tumor, although such selective conditions may also bias clonal representation [39]. All studies relied on core methodologies—namely, enzymatic digestion, 3D culture in Matrigel, and the use of stem cell–supportive media [40,41]. Collectively, these studies indicate that gastric PDO establishment is technically feasible across different patient-derived specimens, while differences in selective culture conditions can substantially influence the representation of malignant clones.

Across these studies, gastric cancer-derived organoids reproduced several morphological, genomic, and molecular features of the primary tumors, although the level of evidence differs among these features. Regarding biological fidelity, Steele et al. reported diverse tumor organoid morphologies and showed preservation of structural and differentiation patterns of the original tumors following transplantation models. Orthotopic transplantation of organoids into murine models was also performed to assess tumorigenicity and in vivo behavior [42]. Together, these findings indicate that organoid morphology can reflect both histopathological and genotype-associated features of gastric cancer, although the extent to which morphology alone captures the full molecular heterogeneity of individual tumors remains uncertain. Nanki and colleagues classified cancer organoids into solid (associated with diffuse-type gastric cancer), glandular (associated with intestinal-type gastric cancer), and mixed forms and showed that *CDH1* knockout converts cystic structures into solid morphologies, directly linking genotype to organoid architecture [39]. Moreover, the biobank of 63 organoid lines from 34 patients established by Yan et al. showed representation of EBV, MSI, CIN, and GS molecular subtypes [38]. Mouse models representing the CIN and GS-WNT subtypes reproduced phenotypic features corresponding to their respective molecular classifications in the study by Seidlitz et al. [41]. While these studies primarily established biological fidelity, evidence for clinical predictive utility remains more limited. Vlachogiannis et al. concentrated on metastatic gastrointestinal cancers, where organoids were derived from multiple biopsies of patients enrolled in prospective clinical trials and were compared with clinical outcomes to evaluate clinical concordance. This study reported high concordance between ex vivo drug responses and clinical responses in the evaluated cohort. Organoids captured temporal tumor evolution and acquired resistance mechanisms. Importantly, intra-patient heterogeneity across different metastatic sites was preserved [40]. However, because this study involved metastatic gastrointestinal cancers rather than gastric cancer exclusively, its findings provide supportive evidence for the broader translational potential of PDOs but cannot by themselves establish gastric cancer-specific clinical utility [38,41]. Collectively, these studies support that PDOs can retain key histopathological, transcriptomic, and genomic features of the original tumors, even after long-term culture or xenotransplantation [38,39,41,42]. To critically assess the translational readiness of patient-derived gastric cancer organoids, the available literature must be stratified according to a formal hierarchy of evidence comprising four distinct tiers: (1) biological fidelity (recapitulation of morphological, histological, and genomic features without clinical comparison); (2) retrospective clinical association (correlation of ex vivo drug sensitivity with outcomes in patients whose treatments were already administered without organoid guidance); (3) prospective clinical concordance (co-clinical observational evaluation where organoids are tested in parallel with prospectively treated patients, but ex vivo results do not direct clinical decisions); and (4) prospective interventional clinical utility (randomized or interventional trials demonstrating improved clinical endpoints when therapy is guided by organoid testing). Table 8A summarizes the current clinical and translational landscape across these evidentiary tiers.

In contrast, evidence for clinical concordance remains limited. In a proof-of-concept analysis by Steele et al., organoid drug response could be compared with clinical response in only two patients [42], and clinical correlation in the biobank of Yan et al. was limited to three patients, whereas high-throughput screening mainly generated hypotheses for translational studies, such as unexpected sensitivities to abemaciclib and the ATR inhibitor VE-822 [38]. Although Vlachogiannis et al. reported high concordance between ex vivo and clinical responses and preserved intra-patient heterogeneity, their cohort consisted of metastatic gastrointestinal cancers rather than gastric cancer exclusively, and therefore provides supportive evidence for the broader translational potential of PDOs but cannot by itself establish gastric cancer-specific clinical utility [40]. Thus, while these studies demonstrate biological fidelity and drug-response heterogeneity, prospective interventional trials (Tier 4) are still required to prove that organoid-guided treatment selection improves patient outcomes over standard care.

A further limitation is that PDOs are often derived from a single biopsy or small tissue fragment and may not fully capture intratumoral heterogeneity. To overcome this, multiple biopsies may be used, although this approach can be ethically and practically challenging and may limit the ability of PDOs to fully represent patient-level tumor heterogeneity [181]. Overall, patient-derived organoids are valuable models for gastric cancer that, in addition to improving our understanding of tumor biology, provide a platform for investigating patient-specific therapeutic responses. The establishment of large PDO biobanks, coupled with prospective clinical studies, could facilitate future translational applications of these models, particularly in potential precision-medicine applications. Beyond sample size, several methodological confounders—such as timing relative to treatment, overgrowth of normal gastric epithelium, and assay turnaround times—further affect clinical implementation, as detailed in Table 8B.

### 6.2. Developmental Biology and Congenital Defects

Gastric organoids derived from human PSCs have provided a unique platform for studying gastric development, modeling congenital disorders, and dissecting signaling pathway functions [4]. By recapitulating the morphological, cellular, and functional characteristics of the human stomach, these organoids enable the investigation of complex developmental processes and inter-germ-layer interactions. Their utility is further enhanced by the integration of precise genetic engineering tools, such as the CRISPR/Cas9 system, and the ability to independently derive and recombine the three germ layers, thereby facilitating in vitro reconstruction of complex aspects of stomach development [4,17,18,35]. Compared to traditional animal models, organoids offer distinct advantages by providing human-specific insights into signaling mechanisms (e.g., species-specific differences in Hedgehog pathway function). Moreover, unlike primary tissue-derived models that rely on variable-quality surgical or cadaveric specimens, organoids can be generated from expandable cell lines, offering greater accessibility and potential scalability, making them well-suited for investigating the role of key genes in cell fate determination [16]. However, this scalability is constrained by lengthy, multistep differentiation protocols, cost, and line-to-line and batch-to-batch variability.

For lineage tracing studies, the generation of genetically engineered reporter cell lines, such as *LGR5*:*eGFP*-expressing hESCs, has enabled the visualization and tracking of *LGR5*^+^ stem cell populations during organoid development [17]. Furthermore, the derivation of GFP-expressing splanchnic mesenchyme (SM) and RFP-expressing enteric neural crest cells from human PSCs, and their recombination with gastric epithelium, has facilitated the examination of cross-talk between germ layers. These studies have demonstrated that ENCCs are essential for gastric mesenchymal expansion and glandular morphogenesis, and that coordinated interaction of ENCCs and SM is necessary for the formation of organized smooth muscle layers and regionally specified gastric epithelium [16].

In pathway analysis, organoids have revealed that epithelial Wnt/β-catenin signaling is required for fundic identity and repression of antral fate. Activation of β-catenin with CHIR99021 in foregut progenitors induces expression of fundus markers (*IRX2*, *IRX3*, *IRX5*) while suppressing *PDX1*, a marker of the antrum [18]. The BMP pathway also plays a critical role in posteriorization; inhibition of BMP signaling by Noggin promotes foregut endoderm fate. ENCCs have been shown to secrete BMP4 and BMP7, which can posteriorize gastric epithelium—an effect that is blocked by BMP inhibition [16,35]. Additionally, the human fundic gastric organoid platform has been applied for functional screening of pathways involved in the differentiation of specific gastric cell types. For instance, transient MEK inhibition in the presence of BMP4 effectively promotes the differentiation of parietal cells, as indicated by the expression of markers such as ATP4A, ATP4B, and GIF, alongside functional acid secretion. Similarly, doxycycline-inducible overexpression of the transcription factor NEUROG3 in hESCs, even under conditions typically suppressive to endocrine lineage commitment (i.e., high EGF), led to differentiation into gastric endocrine cells [18].

Moreover, advances such as the “organoid-on-a-chip” platform have further enhanced physiological relevance by introducing features like luminal flow and externally imposed, pump-driven rhythmic deformation, thereby enabling dynamic assessment of gastric functions and responses to pharmacological agents in vitro [34].

Organoids have also become important tools for investigating the developmental basis of congenital diseases and analyzing the functional consequences of genetic variants. By reconstructing aspects of human development in a three-dimensional context, these systems allow the study of congenital disorders in a context with human tissue architecture. Genetically, CRISPR-based approaches have also enabled the introduction of patient-specific mutations into healthy stem cell lines or the correction of mutations in patient-derived lines, thus validating the causality of variants in congenital diseases [182].

In the case of congenital pyloric conditions, specific genetic mutations have been associated with pyloric atresia, a congenital obstruction or restriction of the gastric outlet. This anomaly is often associated with junctional epidermolysis bullosa, and key risk genes have been identified, including *PLEC1*, *ITGA6*, and *ITGB4* [182,183,184]. Overall, hPSC-derived organoids provide a suitable platform to study the functional effects of these mutations and better understand the developmental mechanisms underlying congenital pyloric restrictions.

In total, hPSC-derived gastric organoids, empowered by genetic engineering and germ-layer recombination strategies, represent a valuable tool for elucidating developmental mechanisms, modeling human congenital gastric disorders, and analyzing germ-layer-specific signaling pathways. These platforms not only overcome key limitations of animal and static primary models but also offer scalable and human-relevant systems for high-resolution in vitro experimentation.

### 6.3. Regenerative Medicine

The ability to generate gastric organoids in vitro from human PSCs has opened new avenues for regenerative medicine research, including the long-term conceptual possibility of organ replacement strategies [17]. Such approaches could, in principle, be relevant for patients lacking functional gastric tissue due to surgery (e.g., gastrectomy) or progressive disease. Patient-derived iPSCs allow organoids with an identical genetic background to be established, and genetic correction could in theory yield isogenic “healthy” organoids. However, gastric replacement using organoids remains an early stage research concept rather than a near-term therapeutic platform, and substantial technical and biological hurdles must be overcome before any clinical application can be considered [7].

Current gastric organoids remain simplified and developmentally immature compared with the adult human stomach; PSC-derived organoids, for example, typically reach only 2–3 mm in diameter [17]. Organoids derived from ASCs [6,19] and from PSCs [17,18] are epithelial in composition, and PSC-derived gastric organoids do not intrinsically develop an abundant mesenchyme [16]; a mesenchymal compartment therefore has to be generated separately and added, as in three-germ-layer gastric organoids [16]. More complex models have started to address this gap (Section 5.5). Three-germ-layer gastric organoids, which combine foregut endoderm, splanchnic mesenchyme, and enteric neural crest cells, form muscle layers, c-KIT^+^ cells, and ganglionic structures resembling enteric plexuses [16], and confinement-based gastric tissues develop smooth muscle and an ENS without exogenous neural crest cells [94]. However, these models still have important limitations: maturation of the muscle and neural components depends on weeks of in vivo engraftment, the identity of ICCs rests on marker expression and localization alone, neuronal activity has only been evoked by external stimulation, and no model includes extrinsic vagal or sympathetic innervation [16,94]. Vascular and immune components have so far been incorporated mainly in non-gastric organoids, such as liver [185] and intestinal [186] organoids, and physiological gastric motility has not been reproduced in any gastric model. Region-specific gastric assembloids have further increased tissue complexity and enabled functional maturation of parietal cells [92]. Whether integrating multiple germ layers and supportive microenvironmental components can ultimately yield transplantable tissue remains to be established.

## 7. Standardization and Quality Control

Establishing standardized quality-control (QC) pipelines is essential for transitioning gastric organoids from exploratory research to reproducible preclinical and translational models [187,188,189,190,191]. Current quality-management approaches rely on different levels of documentation and can be categorized into three distinct tiers: formally established consensus standards, recommended laboratory practices, and methodological aspects that remain insufficiently standardized.

### 7.1. Formally Established Consensus Standards

Official consensus standards have recently been formulated specifically for human gastric cancer organoids [188] and normal human gastric organoids [187], supplemented by broader cross-tissue quality frameworks [189,191]. Within these gastric-specific consensus documents, successful establishment is defined by continuous subculture capacity—requiring at least three passages for tumor organoids and two passages for healthy-donor organoids while preserving phenotypic morphology and epithelial integrity [187,188]. To authenticate donor origin and exclude cross-contamination, these standards mandate short tandem repeat (STR) profiling matched against the parental tissue [187,188]. However, STR matching confirms donor identity but does not establish malignant purity, because it cannot distinguish tumor organoids from overgrowing normal epithelium of the same donor; orthogonal genomic analyses (e.g., targeted sequencing or copy-number profiling) or histopathological confirmation are therefore required [39]. For tumor organoids, consensus guidelines recommend confirming genomic concordance by screening for primary driver mutations (e.g., *TP53*, *CDH1*, *KRAS*, or *PIK3CA*) detected in the original patient tumor [188]. For healthy gastric organoids, standards stipulate confirmation of a normal diploid karyotype, evidence of proliferative activity based on expression of the proliferation marker gene *MKI67*, and verification of lineage-specific markers (including MUC5AC, ATP4B, PGC, MUC6, and CHGA) alongside *LGR5* expression for antral lineages [187]. Furthermore, post-thaw biobanking criteria require documentation of cryopreservation parameters and a recovery threshold of at least 50% viable organoids capable of subsequent passaging [187,188]. These criteria define minimum requirements for establishment, identity, and recovery; they do not by themselves establish suitability for drug-response testing or clinical deployment, for which the assay-level practices described below apply.

### 7.2. Recommended Laboratory Practices

Operational practices that lack formal cross-consortium ratification but represent recommended laboratory methodologies have predominantly emerged from published screening protocols and optimization studies [190,192]. For medium- and high-throughput drug testing, measuring intracellular ATP levels via luminescence is the most widely adopted surrogate readout for cell viability [190,192]. Recommended assay parameters typically include the use of opaque-walled microplates to prevent optical cross-talk, dynamic drug-exposure windows ranging from 72 h to 5 days, and serial dilution ranges comprising at least 6 to 9 concentrations to ensure reliable curve-fitting for IC50 and relative AUC calculations [190,192]. Protocols also commonly recommend a minimum of three technical replicates per condition and, where feasible, independent biological replicate runs to control for plating variability [190,192].

### 7.3. Methodological Aspects Lacking Standardization

Despite these foundational standards and operational protocols, several key methodological areas remain insufficiently standardized across laboratories:

- Quantitative thresholds for normal epithelial overgrowth: Although selective media strategies (such as Wnt or EGF withdrawal and Nutlin-3 exposure) enrich for malignant clones [39], existing consensus standards lack a defined quantitative cutoff (e.g., based on mutant allele frequency or deep sequencing) to formally exclude or score non-malignant epithelial contamination [187,188].
- Upper passage limits for therapeutic assays: While consensus standards mandate recording passage numbers [187,188], no universally accepted upper passage limit has been defined to prevent confounding from culture-acquired genetic drift or epigenetic alteration.
- Quantitative 3D morphological metrics: Current morphology criteria in consensus standards remain predominantly descriptive (e.g., cystic versus bud-like structures) [187,188], and standardized automated image-analysis metrics for 3D architecture are not yet routinely implemented.
- Inter-donor cohort replication: While guidelines define assay-level replicates [190,192], there is no consensus on the minimum number of independent patient-derived lines required to demonstrate the predictive validity of an ex vivo biomarker.

Clarifying the distinction between formally adopted consensus benchmarks, recommended operational protocols, and unresolved technical variables is critical for establishing reproducible workflows in translational gastric organoid research.

## 8. Conclusions, Limitations and Future Perspectives

Gastric organoids have emerged as an accessible and physiologically relevant model for investigating gastric stem cells and differentiated lineages within a controlled microenvironment. PSC-derived gastric organoids provide a useful system for exploring stomach development, congenital abnormalities, and hereditary gastric cancer.

Patient-derived gastric organoids, particularly from individuals with gastric cancer, can serve as valuable tools for drug discovery. Given that the presence of parietal cells is dependent on mesenchymal interactions, elucidating the relationship between *H. pylori* infection, mesenchymal cells, and parietal cell populations may offer critical insights into the early stages of metaplastic transformation in gastric tissue. Notably, the depletion of parietal cells is a hallmark of metaplastic progression, highlighting the potential of gastric organoids in uncovering previously unknown mechanisms underlying *H. pylori*-induced pathogenesis. Notably, these applications differ in their level of maturity. Developmental modeling, epithelial infection models, and tumor organoid biobanks are best supported by gastric data and can be considered established, whereas immune and stromal co-culture, organoid-on-a-chip systems, neuromuscular models, and the concordance between organoid and patient responses remain emerging. Organoid-directed therapy, transplantable tissue, fully defined Matrigel-free culture, and AI-based prediction of drug response remain experimental (Figure 4).

Despite the mentioned advances, gastric organoid models still have several important limitations that constrain their physiological fidelity and clinical translation. Conventional epithelial organoids lack stromal, immune, neural, and vascular components; PSC-derived organoids contain only a limited mesenchymal population, and immune and vascular components are absent from most models, which limits the ability to fully recapitulate the multicellular gastric microenvironment. In addition, muscle and neural models currently rely largely on in vivo engraftment, while variable maturation and inter-model variability can affect reproducibility and interpretation. The use of Matrigel, which is tumor-derived and shows batch-to-batch variability, together with differences in culture protocols and growth conditions, remains a major obstacle to standardization. Although tissue-derived ECM hydrogels have shown initial feasibility in gastric organoids, including a direct comparison with Matrigel (Section 5.2, Table 4), broad and standardized validation across laboratories, passages, lineages, and applications is still lacking before they can reliably replace Matrigel. The relatively high cost and technical complexity of advanced organoid systems further limit their scalability, and the clinical role of patient-derived organoids remains investigational, since concordance with patient response has been reported mainly in small, largely retrospective cohorts.

Future studies may expand the translational potential of gastric organoids by validating defined and tissue-derived matrices in gastric organoids, including long-term culture and parietal cell differentiation, and by incorporating acid-secreting parietal cells into more physiologically complex co-culture systems. The development of gastric–intestinal co-culture platforms that reproduce aspects of gastrointestinal physiology, including luminal conditions and tissue–tissue interactions, may provide opportunities for investigating the delivery and stability of targeted therapeutics and nanomedicines under more physiologically relevant conditions. Standardized reporting of culture conditions and quality-control criteria, together with prospective studies testing whether organoid-guided treatment selection improves outcomes, will also be required. These approaches remain prospective and will require further experimental validation before their clinical applicability can be established.

## Figures and Tables

**Table 1:** Factors used in gastric organoid protocols and their developmental rationale.

Factor	Role in Development	Where It Is Used	Why It Is Added
Activin A	Nodal, a TGF-β family member, controls DE formation [9]	PSC protocol, DE induction (days 0–3) [17]	Induces Nodal signaling to direct PSCs toward DE
Noggin	BMP suppresses SOX2; early BMP inhibition is needed for esophageal and gastric differentiation [18]	PSC protocol, posterior foregut and antral induction [17,18]; ASC medium [19]	Inhibits BMP4 to promote foregut fate (PSC) and to prevent stem cell differentiation (ASC)
FGF4, RA, CHIR99021	FGF, Wnt, and RA pattern the gut tube; *Raldh2-null* embryos show stomach defects [10,20]	PSC protocol, posterior foregut specification (days 3–6) [17].	Posterior foregut specification
FGF10	FGF10/FGFR2 regulates epithelial growth and glandular stomach development [21].	Fundic PSC protocol (from day 20) [18]; mouse PSC and ASC media [19,22].	Supports epithelial growth [21]
BMP4 + MEK inhibitor (PD0325901)	—	Fundic PSC protocol, transient (days 30–32) [18].	Promotes parietal cells (*ATP4A*, *ATP4B*, *GIF*, acid secretion) [18].
RA, Noggin, EGF	RA as above	PSC protocol, antral organoids [17]	Directs differentiation toward antral cell types (mucous neck, pit, G cells); EGF affects NEUROG3 and endocrine cells [17]
SHH, DKK1, Noggin	SHH activation with Wnt inhibition induces primitive tubular structures resembling the early stomach [22]	Mouse PSC protocol, embryoid body stage [22]	—
WNT3A, R-spondin1	Wnt maintains stem cell niche [6]	ASC medium [6,19]	Essential for formation and expansion; R-spondin inhibits RNF43 and ZNRF3 and increases Frizzled at the membrane [19,23]
Gastrin	—	Mouse ASC medium with FGF10, EGF [19]	Stimulates growth and differentiation
TGF-β inhibitor	—	Human ASC medium [6]	Optimal growth and longer organoid lifespan [6]

Abbreviations: DE, definitive endoderm; PSC, pluripotent stem cell; ASC, adult stem cell; BMP, bone morphogenetic protein; FGF, fibroblast growth factor; FGFR, fibroblast growth factor receptor; RA, retinoic acid; MEK, mitogen-activated protein kinase kinase; EGF, epidermal growth factor; SHH, sonic hedgehog; DKK1, Dickkopf-related protein 1; TGF-β, transforming growth factor-beta; RNF43, ring finger protein 43; ZNRF3, zinc and ring finger 3.

**Table 2:** (**A**). Systematic comparison of PSC-derived gastric organoid models: cell sources, lineage reconstitution, and methodological constraints. (**B**). Methodological assessment of ASC- and patient-derived gastric organoid models (PDOs): culture strategies, lineage fidelity, and in vitro limitations.

(**A**)					
**Study**	**Cell Source & Platform**	**Timeline**	**Key Lineages & Markers**	**Key Findings**	**Limitations**
McCracken et al., 2014 [17]	Human PSCs (ESCs/iPSCs); 3D Matrigel dome	~34 days (multistep DE → posterior foregut → antral fate)	Epithelial lineages: Pit, mucous neck, enteroendocrine (G cells, NEUROG3^+^), LGR5^+^ stem cells	Recapitulated embryonic stomach development and foregut patterning; demonstrated epithelial hyperproliferation in response to luminal *H. pylori* microinjection.	Epithelial-only; lacks smooth muscle, vasculature, and enteric innervation; dependence on xenogeneic Matrigel; labor-intensive microinjection for infection assays.
McCracken et al., 2017 [18]	Human PSCs (ESCs/iPSCs); 3D Matrigel dome	~34–40 days (transient MEK inhibition + BMP4 days 30–32)	Fundic epithelial lineages: Parietal cells (ATP4A+, ATP4B+, GIF+), chief cells, pit/neck cells	Identified Wnt/β-catenin signaling as essential for fundic vs. antral specification; established pharmacological protocol (MEKi + BMP4) enabling functional acid secretion.	Lacks stromal microenvironment; incomplete parietal cell maturation compared to in vivo adult tissue; low efficiency of mature parietal cell differentiation per organoid.
Lee et al., 2018 [34]	Human PSC-derived gastric organoids; PDMS microfluidic chip	~34 days PSC organoid differentiation + chip microfluidic loading	Antral gastric epithelium; central hollow lumen	Established continuous microfluidic luminal perfusion and pump-driven rhythmic mechanical deformation; enabled dynamic fluorophore visualization and delivery modeling.	Lacks intrinsic neuromuscular circuitry; rhythmic deformation is externally imposed by a peristaltic roller pump, not autonomous peristalsis; very low experimental throughput.
Broda et al., 2019 [35]	Human PSCs (ESCs/iPSCs); 3D Matrigel dome	34–45 days defined protocol	Region-specific gastric epithelium (antral vs. fundic markers: IRX2, IRX3, IRX5 vs. PDX1)	Defined the concentration- and timing-dependent threshold of canonical Wnt activation determining fundic vs. antral lineage commitment.	Epithelial-predominant; protocol requires intensive, costly growth factor regimens; batch-to-batch line differentiation variability.
Eicher et al., 2022 [16]	Human PSC-derived DE + PSC-derived splanchnic mesenchyme + PSC-derived ENCCs; assembly + in vivo engraftment	In vitro multi-lineage assembly (~28–35 days) followed by 8–10 weeks murine kidney capsule engraftment	Three-germ-layer stomach tissue: Glandular epithelium, muscularis mucosae/externa (α-SMA^+^), c-KIT+ ICCs, myenteric/submucosal enteric plexuses (nNOS^+^, ChAT^+^)	Demonstrated that ENCCs drive mesenchymal expansion and coordinated smooth muscle morphogenesis; tissue strips showed TTX- and L-NAME-sensitive, EFS-evoked contractile waves.	Maturation of organized muscle layers and neural plexuses strictly depends on prolonged in vivo transplantation; lacks functional slow-wave electrophysiological recordings; lacks extrinsic vagal/sympathetic innervation.
Krishnamurthy et al., 2022 [36]	Patient-derived iPSCs (harboring PDX1 mutations); 3D Matrigel dome	~34 days direct differentiation	Gastric and intestinal-like epithelial lineages; enteroendocrine cells	Uncovered that human PDX1 loss-of-function drives aberrant gastric/intestinal metaplastic transitions and profound depletion of enteroendocrine subsets.	Does not model systemic or paracrine metabolic/stromal interactions; limited to early developmental phenotype modeling in an epithelial context.
(**B**)					
**Study**	**Cell Source**	**Culture Matrix and Platform**	**Key Lineages and Markers**	**Key Finding**	**Limitations**
Barker et al., 2010 [19]	Murine pyloric antrum crypts	3D Matrigel dome (Wnt3A, R-spondin1, Noggin, EGF, FGF10)	Single-layered epithelial organoids; Lgr5^+^ stem cells, pit cells, mucous neck cells (MUC6^+^, PGC^+^)	First proof-of-concept for long-term (>1 year) adult gastric stem cell self-renewal without mesenchymal feeder cells.	Lacked parietal cells; murine tissue only; entirely epithelial (devoid of immune, vascular, and stromal compartments).
Katano et al., 2013 [37]	Neonatal mouse glandular stomach	Air–liquid interface (ALI) in type I collagen gel	Polarized epithelial monolayer (MUC5AC^+^) surrounded by an outer layer of myofibroblasts	Maintained viability of gastric epithelial cells and native myofibroblasts concurrently for >80 days.	Low throughput; technically challenging Transwell setup; limited validation in human tissue; restricted access to the apical epithelial surface.
Bartfeld et al., 2015 [6]	Human primary gastric tissue (surgical/biopsies)	3D Matrigel dome (supplemented with TGF-β inhibitor, Wnt, R-spondin)	Differentiated into gland-type (*CGB*^+^) vs. pit-type (MUC5AC^+^) organoids; LGR5^+^ progenitors	Established the first long-term, genetically stable human adult gastric organoid culture; recapitulated epithelial innate immunity to *H. pylori.*	Epithelial-only; microinjection into tiny cystic lumens is labor-intensive and has low throughput; lacks stromal/immune cells.
Schlaermann et al., 2016 [5]	Human gastric surgical tissue	3D Matrigel spheroids converted to 2D collagen-coated monolayers	Polarized epithelial monolayers with intact apical-basal junctions (ZO-1^+^, E-cadherin^+^)	High-throughput conversion of 3D organoids into 2D monolayers for direct apical access without microinjection.	Monolayers lose 3D crypt architecture and self-renewing stem cell dynamics; infected cells detach/succumb within 24–48 h.
Yan et al., 2018 [38]	Human primary gastric adenocarcinoma & normal mucosa	3D Matrigel dome	63 lines capturing major TCGA subtypes (EBV, MSI, CIN, GS)	Established a comprehensive living biobank for subtype-specific compound screening; demonstrated viral stability in EBV lines.	Did not use selective media to prevent normal epithelial overgrowth; variable line persistence across subtypes.
Nanki et al., 2018 [39]	Human gastric cancer resections, biopsies, and ascites	3D Matrigel with selective growth-factor withdrawal	Solid, glandular, and mixed tumor morphologies; preserved driver mutations (*TP53*, *CDH1*)	Introduced niche-factor withdrawal (EGF/FGF10, Nutlin-3) to systematically suppress normal cells and enrich tumor clones.	Selective culture media can artificially bias clonal dominance; normal-like organoid overgrowth still occurred in 9 of 46 sequenced lines.
Vlachogiannis et al., 2018 [40]	Fresh metastatic gastrointestinal biopsies	3D Matrigel dome	Recapitulated histological and molecular characteristics (>90% somatic variant allele concordance)	Demonstrated ex vivo feasibility of testing targeted agent libraries against metastatic gastrointestinal tumors within ~2 weeks.	Mixed GI cancer cohort (mostly colorectal; only 4 gastroesophageal cases); lack of immune/stromal microenvironment.
Seidlitz et al., 2019 [41]	Surgical resection (gastric and GEJ adenocarcinoma)	3D Matrigel dome	Preserved primary histopathology and targeted genomic alterations (CIN and GS models)	Refined mechanical isolation of tumor patches to minimize healthy gland contamination; established subtype-specific drug profiles.	Small number of lines characterized in depth; lacks native tumor stroma; normal gland overgrowth remains a risk without genetic selection.
Steele et al., 2019 [42]	Human gastric cancer resections	3D Matrigel dome	Recapitulated patient tumor histology; tumorigenic in subcutaneous and orthotopic murine xenografts	Demonstrated parallel in vitro drug sensitivity testing alongside orthotopic in vivo tumorigenicity assays.	Low establishment and persistence in diffuse/signet-ring cell carcinoma (SRCC); lack of immune microenvironment components.
Giobbe et al., 2021 [43]	Pediatric, fetal, and adult human gastric tissues	3D Matrigel & apical-out polarity reversal	Stage-dependent ACE2 receptor expression and gastric epithelial differentiation	Demonstrated age-dependent gastrointestinal tropism of SARS-CoV-2; developed polarity-reversed organoids for viral access.	Epithelial-only; lacks systemic immune response; polarity reversal protocols add technical complexity.
Aguilar et al., 2022 [44]	Human gastric tissue units	3D Matrigel dome	Highly differentiated pit cells (GKN1^+^, GKN2^+^, PSCA^+^) vs. deeper gland cells	Revealed bacterial tropism of *H. pylori* to differentiated surface pit cells driven by host-derived urea chemotaxis.	Short-term host–pathogen interaction window; lack of inflammatory cell recruitment and vascular components.

Abbreviations: PSC, pluripotent stem cell; ESC, embryonic stem cell; iPSC, induced pluripotent stem cell; DE, definitive endoderm; ENCC, enteric neural crest cell; ICC, interstitial cell of Cajal; EFS, electrical field stimulation; TTX, tetrodotoxin; L-NAME, N(ω)-nitro-L-arginine methyl ester; MEKi, MEK inhibitor; PDMS, polydimethylsiloxane. Abbreviations: ASC, adult stem cell; PDO, patient-derived organoid; GC, gastric cancer; GEJ, gastroesophageal junction; ALI, air–liquid interface; TCGA, The Cancer Genome Atlas; CIN, chromosomal instability; MSI, microsatellite instability; EBV, Epstein–Barr virus; GS, genomically stable.

**Table 3:** Systematic comparison of principal culture platforms and matrices used in gastric organoid research across performance metrics.

Culture Platform or Matrix	Throughput & Automation Potential	Batch Reproducibility & Standardization	Immune & Stromal Suitability	Scalability & Translational Readiness	Principal Advantages	Principal Limitations	Key Applications
Matrigel-Based 3D Dome Culture [5,6,55,56,57,58]	Moderate: Standard 24- and 48-well plate formats; automated liquid handler dispensing into 96/384-well plates is technically challenging due to temperature-dependent gelation.	Low to Variable: High lot-to-lot variability in protein concentration, growth factor composition, and matrix stiffness; murine sarcoma origin introduces unstandardized biological cues.	Poor: Yields predominantly epithelial-only organoids; dense ECM dome restricts physical infiltration and passive migration of co-cultured exogenous immune cells (e.g., T cells, PBMCs).	Moderate: Labor-intensive manual passaging; high cost of GMP-grade basement membrane extracts; limited utility for clinical cell transplantation due to xenogeneic components.	Supports efficient 3D self-organization, apical-basal polarity, and long-term stem cell maintenance; historical reference standard with standardized growth factor protocols.	Undefined batch composition; diffusion limits restrict organoid diameter (≤200–300 μm without necrosis); lack of native stromal and immune microenvironment.	Epithelial lineage differentiation, basic disease modeling, large-scale PDO biobanking, medium-throughput drug screening.
Air–Liquid Interface (ALI) [37,59,60,61]	Low: Restricted to specialized Transwell filter inserts; manually demanding; incompatible with standard automated high-throughput screening robotic platforms.	Moderate: Collagen matrices offer more consistent biochemical properties than Matrigel, but culture outcome depends on stromal cell viability and operator technique.	Moderate to High: Retains endogenous stromal myofibroblasts and enables co-culture with primary immune cells via apical or basolateral medium compartments.	Low: Technically intricate; high labor overhead per well; low tissue yield relative to culture footprint limits scalable biomanufacturing.	Apical air exposure dramatically enhances oxygenation and epithelial differentiation (e.g., foveolar mucous cells); supports prolonged viability (>80 days).	Limited validation in human gastric tissue; restricted apical access for biochemical sampling; imaging through porous transwell membranes is challenging.	Mucosal barrier physiology, chronic epithelial-stromal interaction studies, long-term non-malignant mucosal homeostasis.
Organoid-Derived 2D Monolayers [5,62,63]	High: Highly compatible with standard 96- and 384-well planar plates, automated plate readers, high-content imaging systems, and robotic liquid handling.	High: Uniform cell seeding density and planar geometry minimize optical and diffusion artifacts; highly reproducible barrier resistance measurements (TEER).	Moderate: Direct apical or basolateral addition of leukocytes and cytokines; lacks 3D architectural stroma but allows precise kinetic measurement of immune cytotoxicity.	High: Continuous and scalable generation by enzymatically dissociating biobanked 3D organoids; cost-effective and rapid turnaround for compound screens.	Direct apical accessibility without microinjection; simplified infection workflows; high optical resolution for live-cell and confocal microscopy.	Complete loss of 3D glandular architecture and crypt-villus spatial gradients; reduced stem/progenitor self-renewal; cultures succumb rapidly (≤24–48 h) to bacterial toxins.	High-throughput drug screening, *H. pylori* adhesion and CagA translocation dynamics, epithelial barrier permeability, innate immune signaling.
Organoid-on-a-Chip (Microfluidics) [34,62,64,65,66]	Very Low to Low: Restricted to microfabricated chips with complex tubing, peristaltic pumps, and specialized pneumatic interfaces; unsuited for high-throughput screening.	Moderate to High: Precisely engineered channel dimensions, continuous laminar flow, and controlled mechanical shear; susceptible to bubble formation and fluidic clogging.	High: Multi-channel compartmentalization allows spatial co-culture of epithelium with endothelium and circulating immune cells under physiological shear stress.	Low: High device fabrication costs; low sample throughput; demanding operational requirements restrict deployment to specialized bioengineering laboratories.	Recapitulates dynamic luminal perfusion, apical acid gradients, shear stress, and pressure-driven cyclic mechanical deformation; bilateral apical/basal access.	High technical complexity; PDMS can absorb hydrophobic small molecules; low experimental throughput; long-term operation prone to channel leakage or disruption.	Mechanobiology, dynamic host–pathogen interactions (e.g., polarized *H. pylori* colonization under flow), oral drug permeability, vascular-immune extravasation.

Abbreviations: ALI, air–liquid interface; ECM, extracellular matrix; GMP, good manufacturing practice; PBMC, peripheral blood mononuclear cell; PDO, patient-derived organoid; PDMS, polydimethylsiloxane; TEER, transepithelial electrical resistance; 2D, two-dimensional; 3D, three-dimensional.

**Table 4:** Comparison of Matrigel and alternative matrices for gastric organoid culture.

Matrix	Composition and Tunability	Reproducibility and Scalability	GMP/Translational Aspects	Evidence in Gastric Organoids	References
Matrigel	Tumor-derived, undefined; limited tunability	Batch variability; widely available	Xenogeneic, tumor-derived	Reference standard	[5,6,55,57,58,67,69]
Decellularized tissue ECM	Tissue-specific, partly characterized; stiffness variable	Batch variation; each batch tested	GMP-compatible pipeline described (porcine); certified clinical-grade manufacture not demonstrated	Growth similar to Matrigel; parietal differentiation incomplete	[70,71,72]
Recombinant ECM proteins	Defined; designable	NR	MP-compatible pipeline described (porcine); certified clinical-grade manufacture not demonstrated	None identified	[68]
Synthetic (PEG/VitroGel)	Fully defined; stiffness, adhesion, degradability tunable	Described as reproducible	Described as potentially GMP-compatible (not demonstrated in gastric organoids); lacks native signals	Preliminary: human gastric organoids maintained in VitroGel with DC recruitment in a short-term chip/co-culture system; no long-term propagation [73]	[70,71,74,75]

Qualitative summary based on the cited studies; no head-to-head comparison of all matrices in gastric organoids has been reported. Evidence for recombinant matrices derives from intestinal organoids; gastric evidence for synthetic hydrogels is limited to a short-term feasibility study [73]. NR, not reported in the studies identified. Abbreviations: ECM, extracellular matrix; GMP, good manufacturing practice; PEG, polyethylene glycol.

**Table 5:** Immune and stromal components in gastric organoid models: evidence, retained cell populations, duration, and limitations.

Platform	Gastric-Specific Evidence	Cells Retained or Added	Duration	HLA Match	Antigen Presentation	Myeloid Cells	Spatial Organization
Epithelial-only PDOs	Biobanks and drug-response studies [38,39,41,42,85,86,87] did not report systematic retention of TME components.	Tumor epithelium (± normal epithelium)	Serial passaging; drug assays within days to ~2 weeks (Table 8A)	Not testable (no immune cells)	Tumor MHC/antigen-processing state not functionally assessable	None	Epithelium in Matrigel; no stromal or immune architecture
Transient carry-over of endogenous cells	Residual stromal fibroblasts persisted in early cultures and interfered with the growth of three SRCC-derived PDOs [87]; endogenous immune or stromal carry-over was not systematically quantified in the gastric PDO studies reviewed	Residual fibroblasts in early cultures; immune-cell carry-over not quantified	Early passages; not systematically quantified across gastric lines	Initially patient-matched by origin; persistence and functional HLA-mediated interactions were not established.	Native APCs not maintained	Not maintained	Lost on dissociation
Added autologous T cells ± DCs/MDSCs	huTGOs ^+^ autologous CTLs (HER2–PD-L1 axis), with MDSCs suppressing killing [82]; *H. pylori*-infected human gastric organoids co-cultured with autologous dendritic cells and cytotoxic T lymphocytes [84]; evidence of infection-related epithelial–immune interactions, rather than validation of a gastric tumor model; (immune–epithelial interaction evidence, not validation of a tumor model)	PBMC-derived CTLs, DCs, MDSCs (expanded ex vivo)	Assay duration 24–72 h; long-term persistence NR	Autologous matching reduces alloreactivity concerns; yield-limited	Tumor-specific recognition depends on T-cell specificity, priming strategy, and functional antigen presentation; antigen presentation by tumor cells and exogenous APCs should be evaluated separately.	DCs/MDSCs only, added exogenously	Cells mixed into Matrigel; not native
Added allogeneic PBMCs/NK cells	6 PMGC organoid lines + pooled PBMCs from 4 donors; cytolytic vs. non-cytolytic lines independent of HLA mismatch; pembrolizumab effect in 1 of 3 PD-L1-high lines [81]; PBMC and NK overlay co-culture with colorectal tumor spheroids, 50:1 (non-gastric) [88]	CD3/CD28-activated T cells, NK/NKT cells	48–72 h [81]	Allogeneic; cytotoxicity was not explained by HLA matching alone, and alloreactivity could not be excluded.	Not assessed; tumor-antigen-specific recognition was not established.	Absent	Co-embedded in Matrigel; infiltration seen, no stroma
CAF co-culture	23 PDOs (12 PCC, 11 non-PCC) co-cultured with patient-derived CAFs: increased organoid growth and chemoresistance [89]	Culture-expanded patient CAFs	Typically, 3–7 days for functional co-culture assays	Not applicable (no immune cells)	Not modeled	None	CAF network around organoids in direct contact [89]; full immune compartment and native stromal architecture not reproduced (design limitation)
ALI tumor-fragment cultures	No gastric tumor ALI study with retained immune cells identified; mouse gastric ALI retained myofibroblasts [37]; shown in other non-gastric solid tumors [90]	Non-gastric: T, B, NK cells, macrophages, fibroblasts [90]	Gastric tumor: NR; mouse gastric stromal ALI > 80 days [37]	Autologous by tissue origin; functional immune interactions not established in gastric tumors.	Native APCs possible; not verified in gastric tissue	Retained in non-gastric tumors [90]	Fragment architecture partly retained; needs sufficient tissue
Assembloids	Patient-derived gastric tumor organoids + autologous stromal subpopulations; higher inflammatory and ECM-remodeling programs vs. organoids alone; altered drug response, with stroma-mediated resistance not consistent across patients [91]	Autologous stromal cells expanded in MSC, endothelial and fibroblast media; no immune cells	~5 days of culture; 72 h drug exposure	Not applicable (no immune cells)	Not applicable (no immune cells)	None	Central epithelial clusters surrounded by stroma; Matrigel needed for aggregation; 1:1:4 (ECM:FM2:organoid) used [91]
Multi-regional epithelial assembloids (non-tumor)	Paediatric fundus/body/antrum organoids self-assembled in collagen I; enhanced parietal-cell maturation and acid secretion; modelled antral foveolar hyperplasia in PMM2-HIPKD-IBD [92]	Epithelial cells only; no stromal or immune cells	10 days of assembloid culture [92]	Not applicable (no immune cells)	Not applicable (no immune cells)	None	Linear fundus–body–antrum tube with shared lumen and gland-like protrusions [92]
Microfluidic systems	Non-malignant gastric organoids + monocyte-derived DCs on chip improved DC migration [73]; epithelium–fibroblast–endothelium prototype [61]; epithelium-only non-malignant chip [65]; in the studies reviewed, no gastric tumor–immune chip validated for ICI response	Epithelium; DCs; fibroblasts/endothelium (differs by system; DC chip and epithelium-only chip are distinct)	Up to 13 days (epithelium-only chip) [65]	Not reported in sources reviewed	T-cell antigen presentation not assessed in sources reviewed	DCs in the gastric DC-chip study; myeloid components not established in the other cited gastric systems.	Compartmented channels with perfusion; low throughput

Evidence from non-gastric tissues is included only to provide methodological context and should not be interpreted as direct validation of these approaches in gastric cancer. NR, not reported in the sources reviewed. Definitions of clinical outcomes used for diagnostic metrics: Schmäche et al. [86], pathological response after neoadjuvant FLOT according to Becker regression grading (responders, grade 1a/1b; non-responders, grade 2/3); Huang et al. [87], ex vivo sensitivity defined as an inhibition rate ≥50% at the plasma steady-state concentration (Css), compared with tumor recurrence or metastasis during disease-free survival follow-up (stage I–III) or progressive disease (stage IV); Vlachogiannis et al. [40], radiological response according to RECIST 1.1, comparing disease control (partial response or stable disease) with progressive disease (primary or acquired resistance). Yan et al. [38], Steele et al. [42], and McDonald et al. [85] reported descriptive comparisons without formal diagnostic thresholds. Abbreviations: ALI, air–liquid interface; APC, antigen-presenting cell; CAF, cancer-associated fibroblast; CTL, cytotoxic T lymphocyte; DC, dendritic cell; ECM, extracellular matrix; GC, gastric cancer; huTGO, human gastric tumor organoid; ICI, immune checkpoint inhibitor; MDSC, myeloid-derived suppressor cell; MSC, mesenchymal stem cell; NK, natural killer; PBMC, peripheral blood mononuclear cell; PCC, poorly cohesive carcinoma; PDO, patient-derived organoid; PMGC, peritoneal metastatic gastric cancer; TME, tumor microenvironment.

**Table 6:** Smooth muscle, ICC, and ENS incorporation across gastric and non-gastric organoid models.

Model	Origin	Organ	Setting	SMC	ICC	ENS	References
Early hAGO/hFGO	Human hPSC	Gastric	In vitro	—	—	—	[17,35]
Gastric organoids with muscularis mucosae	Human hiPSC	Gastric	In vitro	M	—	—	[93]
Three-germ-layer thAGO/thFGO	Human hPSC	Gastric	In vitro assembly + in vivo engraftment	F	M	F	[16]
Confinement-based gastric CCS	Human hPSC	Gastric	In vitro assembly + in vivo engraftment	F	M	F	[94]
Gastric organoid-on-chip	Human hPSC	Gastric	In vitro	—	—	—	[34]
Mouse ESC gut-like organoids	Mouse ESC	Non-gastric	In vitro	F	E	M	[95]
Chick hindgut contractile organoids	Chick embryo	Non-gastric	In vitro	F	E	—	[96]
Bioengineered intestinal muscularis	Mouse/Human tissue	Non-gastric	In vitro	F	F	F	[97]
HIO + ENS	Human hPSC	Non-gastric	In vitro assembly + in vivo engraftment	F	F	F	[79]

Evidence levels: —, not reported; M, marker-based or structural evidence; F, functional evidence; E, direct electrophysiological or optical evidence. Levels reflect the highest evidence reported in the cited study. Abbreviations: SMC, smooth muscle cell; ICC, interstitial cell of Cajal; ENS, enteric nervous system; hPSC, human pluripotent stem cell; hiPSC, human induced pluripotent stem cell; ESC, embryonic stem cell; hAGO, human antral gastric organoid; hFGO, human fundic gastric organoid; thAGO, three-germ-layer human antral gastric organoid; thFGO, three-germ-layer human fundic gastric organoid; CCS, condensed cellular spheroid; HIO, human intestinal organoid; M, marker-based or structural evidence; F, functional evidence; E, direct electrophysiological or optical evidence.

**Table 7:** Approaches for infecting gastric organoids and organoid-derived models with *H. pylori*.

Infection Approach	Apical Accessibility	Maintains 3D Architecture	Throughput	Duration	Quantitation	Major Limitation	References
Microinjection	Via the lumen	Yes	Organoids injected individually (~0.2 µL each)	1–24 h	CFU, qPCR, microarray, Western blot, imaging (IF/confocal, EM, H&E)	Labor-intensive compared with 2D infection	[5,6,134] ^a^
Mechanical disruption/shearing	Direct (transient access during luminal fragmentation)	Partially (disrupted fragments reseal and recover 3D cystic lumen upon re-embedding)	Moderate (batch-level fragmentation by pipetting)	2–6 h (typically ~4 h)	CFU, qPCR, IF, Western blot	Lacks selective control over apical vs. basolateral exposure; heterogeneous fragment sizes; uneven bacterial load	[135,136]
2D monolayer	Direct	No	Easier to handle; 3D spheroids serve as an unlimited cell source	3–22 h	Microarray, Western blot (CagA translocation/phosphorylation), phase-contrast microscopy	Infected cultures succumb within 24 h; loss of 3D architecture	[5,137]
ALI-derived polarized monolayers (mucosoid) ^b^	Direct (applied on top of filters)	No (polarized monolayer)	Transwell inserts in 24-well plates (200,000–250,000 cells per insert)	6 h–3 days; long-term 2–4 weeks	qPCR (bacterial load), qRT-PCR, microarray, Western blot (CagA), confocal IF, TEM, CFU	Non-homogeneous infection due to the mucus layer; bacteria undetectable at 4 weeks; regular mucus removal needed	[137]
Organoid-on-a-chip (patterned, bilateral access)	Apical and basal	Patterned 2.5D–3D	NR (microfabricated device)	2–6 days	CFU, scRNA-seq, confocal IF, live imaging	Requires microfabricated devices; local epithelial disruptions after ~13 days	[65]

Entries are as reported in the cited studies. ^a^ Murine organoid in [134]; the other studies used human cells. ^b^ Cultures derived either from freshly isolated glands or from organoids. Abbreviations: 3D, three-dimensional; 2D, two-dimensional; 2.5D, two-and-a-half-dimensional; ALI, air–liquid interface; CFU, colony-forming unit; NR, not reported.

**Table 8:** (**A**). Comparison of patient-derived organoid (PDO) studies in gastric, esophagogastric and other gastrointestinal cancers: denominators, evidence level, and concordance with clinical response. (**B**). Comparison of PDO studies: specimens, drugs, confounders, timing, blinding, and limitations relevant to clinical scalability.

(**A**)							
**Study**	**Specimens**	**PDOs Established**	**PDOs Drug-Tested**	**Clinically Evaluable Pairs (Ex Vivo vs. Clinical)**	**Evidence Level and Design**	**Concordance Metric**	**Turnaround (Measured vs. Retrospectively Estimated)**
Yan 2018 [38]	35 patients initially sequenced; 34 analyzed (1 excluded due to cross-sample contamination); denominator of total attempted tissue specimens not reported in primary study (NR); cohort comprises consecutive successfully established lines.	63 lines established (46 tumor-derived and 17 normal); per-group attempted specimen denominators not reported in primary study (NR).	9/46 cancer lines from 7 patients (37-compound screen); 10 additional lines tested with ATR inhibitors.	3/7 drug-tested patients with matched clinical outcome data (GX024, GX034, GX068).	Biological fidelity (preclinical biobank); exploratory clinical correlation (*n* = 3)	Qualitative descriptive correlation (*n* = 3): ex vivo 5-FU/cisplatin sensitivity matched partial remission in 2 patients (GX024, GX034); ex vivo 5-FU resistance matched disease progression in 1 patient (GX068). No formal diagnostic performance metric or predefined viability cut-off reported.	Measured assay duration < 2 weeks (screen only); time to establishment NR
Nanki 2018 [39]	101 specimens attempted (42 before and 59 after protocol standardization).	67 lines from tumor tissue (23/42, 54.7% before; 44/59, 74.6% after standardization); of 46 sequenced lines, 37 confirmed gastric cancer organoids (from 36 patients) and 9 were normal-like overgrowth lines.	Selected subsets: 16 Wnt-independent lines tested with C59; 5 *ERBB2*-amplified lines tested with a pan-*ERBB* inhibitor; *1 MET*-amplified line tested with crizotinib.	0 (none; purely preclinical functional genomics without clinical response correlation).	Biological fidelity (functional genomics); no clinical comparison	Not applicable (genotype–phenotype study)	NR
Vlachogiannis 2018 [40]	110 metastatic biopsies from 71 heavily pretreated patients across 4 trials (mixed GI cohort: colorectal, gastroesophageal, cholangiocarcinoma).	23 PDO lines successfully expanded and profiled; 70% success in biopsies with cellularity ≥2+; overall attempted-biopsy denominator and gastroesophageal-specific derivation rate not reported in primary study (NR).	23 lines tested against a 55-compound library.	21 matched comparisons (16 metastatic CRC, 4 metastatic gastroesophageal cancer, 1 cholangiocarcinoma).	Prospective clinical concordance (co-clinical; organoid results did not guide treatment)	Sens 100%, spec 93%, PPV 88%, NPV 100% for ex vivo vs. clinical response (21 matched comparisons; mixed GI cohort, 4 gastroesophageal)	Reported assay duration ~2 weeks (screen only); time from biopsy to result NR
Seidlitz 2019 [41]	20 pts attempted-specimen denominator not stated	20 cultures initiated; stable long-term expansion rate not reported in primary study (NR).	4 lines characterized in depth for chemotherapy and targeted-therapy sensitivity.	0 (none; patient clinical response not assessed).	Biological fidelity (preclinical modeling)	None	NR
Steele 2019 [42]	10 primary tumor resections attempted.	7/10 (70.0%) lines initially formed (huTGO1–7); 3 failed to grow (1 diffuse, 2 signet-ring); huTGO3 was lost after passage 4 and huTGO5 failed to persist.	5/7 lines tested with chemotherapy (EOX) or mubritinib.	2/5 pairs with matched histopathological regression data (huTGO4, huTGO7).	Biological fidelity (preclinical pilot); exploratory clinical comparison (n = 2)	Descriptive qualitative comparison (n = 2 pairs): huTGO7 showed high ex vivo chemosensitivity corresponding to complete pathological response; huTGO4 showed partial ex vivo response corresponding to pathological non-response. No formal diagnostic cut-off defined.	Projected, not measured: testing within 3 days of surgery, results in 5–6 days (not prospectively validated)
McDonald 2023 [85]	19 patients biopsied via EGD; 15/19 (78.9%) were technically adequate (2 lacked malignancy, 2 contaminated).	8/15 (53.3%) technically adequate biopsies established organoids: intestinal 3/3, mixed 2/2, mucinous 1/1, diffuse 1/4, signet-ring 0/2, and poorly differentiated 1/2.	2/8 lines successfully expanded for drug testing (hGT21, hGT25).	2/2 pairs with available patient outcomes.	Exploratory clinical comparison (n = 2); pilot/proof of concept, prospective vs. retrospective design not stated	Uncontrolled descriptive association (n = 2): both patients received regimens matching the best ex vivo sensitivity (ECF for hGT21, FLOT for hGT25) and remained disease-free approximately 3 years after surgery. No comparator group; sensitivity and specificity not calculable.	Reported laboratory processing time (not clinical result delivery): PDO within 24 h; drug testing of two lines within 12 days of biopsy
Schmäche 2024 [86]	132 patients enrolled; 120 biopsies received from treatment-naïve patients with esophagogastric adenocarcinoma (1 excluded due to incorrect diagnosis, 11 biopsies not received); among the 26 FLOT-analyzed patients, 17 had GEJ tumors (65%) and 9 had stomach tumors (35%).	73/120 biopsies received successfully expanded (60.8%); 47/120 (39.2%) did not yield expandable organoids. The 73 successful expansions included 9 normal gastric organoids.	26/73 successfully expanded PDOs tested with FLOT chemotherapy (13 in the exploratory cohort and 13 in the independent validation cohort).	26 patients with matched PDO drug-response and pathological regression data after neoadjuvant FLOT; 50 patients in the broader cohort had pathological response assessed.	Prospective clinical concordance (OPPOSITE co-clinical trial): prospective collection; exploratory cohort (n = 13) then independent validation cohort (n = 13); turnaround estimated retrospectively; not interventional	Exploratory (n = 13: 8 responders, 5 non-responders by Becker): Kendall τ 0.694 (FLOT); AUC_rel threshold 0.559 set here. Independent validation (n = 13: 3 responders, 10 non-responders): 12/13 correctly classified; sens 90%, spec 100%, accuracy 92% for PDOs tested at least twice (PPV/NPV NR)	Retrospectively estimated time to first potential FLOT result (25 of 26 FLOT-tested pts): median 20 days (direct processing, *n* = 18) vs. 62 days (shipped biopsies, *n* = 7; *p* = 0.045); not a measured result delivery
Huang 2026 [87]	27 surgical or biopsy specimens attempted from patients with locally advanced gastric cancer at a single center in 2022.	17/27 (63.0%) successfully established; by Lauren type: intestinal 7/8, mixed 8/11, diffuse 2/8. The 10 failures were attributed to contamination, tissue hardness, or necrosis; counts by reason were not reported (NR).	13/17 established PDO lines tested (76.5%); 3 signet-ring cell carcinoma (SRCC)-derived lines stopped growing owing to stromal fibroblast binding, and 1 additional line was lost for an unstated reason; the 13 tested lines included 4 SRCC-derived and 9 non-SRCC-derived PDOs.	13 patients had clinical follow-up (10 stage I–III patients evaluated for DFS and 3 stage IV patients evaluated for PFS); 11 were included in the binary concordance analysis. GC-001 and GC-004 were excluded because they did not receive postoperative therapy.	Retrospective clinical association (design not labeled; PDO results compared with regimens already received); not interventional	Sens 85.7%, spec 100%, accuracy 90.9% (n = 11 matched cases, Figure 5D); DFS log-rank *p* = 0.044 (6 sensitive vs. 4 resistants; median follow-up 21.0 vs. 18.8 months; 2 recurrences, both in the resistant group); stage IV PFS: 1 sensitive (24 months) vs. 2 resistant (median 2.7 months)	NR; assay workflow only (4 days seeding + 4–6 days drug exposure; establishment time not included)
(**B**)							
**Study**	**Specimen Source**	**Drugs Tested**	**Tumor vs. Normal Epithelial Overgrowth**	**Timing Relative to Patient Therapy**	**Blinding**	**Heterogeneity Handling**	**Principal Limitation**
Yan 2018 [38]	Tumor, dysplastic, normal and lymph-node metastasis tissue (surgical)	37 approved/trial compounds (e.g., napabucasin, abemaciclib, VE-822)	Monitored by IHC/genomic profiling; no selective media used to inhibit normal outgrowth	Surgical resection; 31/34 pts treatment-naïve at surgery, 3 pts received chemotherapy before surgery (including GX068)	NR	Primary tumor vs. lymph-node metastasis organoids compared; varying heterogeneity	Not designed as a formal clinical concordance study; clinical correlation was limited to three patients; cohort limited to consecutive cases with successfully established organoids, so attempted-specimen denominators are unavailable.
Nanki 2018 [39]	Surgical resection, biopsy, and ascites	Niche-factor (Wnt/R-spondin) withdrawal; Wnt-targeting therapy (xenograft)	Actively controlled via niche-factor withdrawal (EGF/FGF10) and Nutlin-3 selection; 9 of 46 sequenced lines were normal-like overgrowth organoids	NA	NA	Divergent genetic/epigenetic routes to niche independence across lines	No clinical concordance; Wnt-targeting tested only in xenografts
Vlachogiannis 2018 [40]	Fresh biopsies of metastases	55-compound library (targeted agents and chemotherapy)	Tumor cell purity confirmed by histopathology and next-generation sequencing (>90% somatic variant allele concordance with metastases)	Biopsies taken prior to trial therapy; heavily pretreated cohort	NR	Assessed across multiple metastatic lesions in select patients	Mixed tumor types (mGOC *n* = 4 of 21 comparisons); sensitive/resistant definition not explicit; small evaluable cohort
Seidlitz 2019 [41]	Surgical resection: gastric and esophagogastric-junction adenocarcinoma (AEG I–III), plus one lung metastasis specimen	5-FU, oxaliplatin, irinotecan, epirubicin, docetaxel; targeted agents (trastuzumab, palbociclib, imatinib)	Protocol optimized (tissue digestion, selection of tumor patches) to minimize carry-over of normal glands; no contamination counts reported	Mixed: a subset of patients received neoadjuvant chemotherapy before resection	NA	Divergent drug responses among the 4 lines analyzed in depth	No comparison with patient response
Steele 2019 [42]	Surgical resection; normal fundic organoids from sleeve gastrectomy (unmatched donors)	Epirubicin, oxaliplatin, 5-FU (EOX); HER2 inhibitor mubritinib; assay readout 24–96 h (flow cytometry cell death) or 48 h	NR	Per Table 1, EOX in 4 of 10 cases (huTGO1, 4, 5, 7; regression grading implies preoperative treatment), no chemotherapy in 2 (huTGO2, huTGO6), treatment unknown in 4	NR	Divergent responses between lines; single line per patient	Very small sample; no immune component; inconsistent statements on line persistence
McDonald 2023 [85]	EGD biopsy, overnight shipping	FLOT, ECF, FOLFIRI, FOLFOX	NR	EGD biopsy; 13/15 evaluable pts received chemotherapy (5 neoadjuvant, 2 adjuvants, 5 palliative), 2 none; biopsy timing relative to therapy and study design (prospective vs. retrospective) not stated	NR	WES concordance between tumor, PDO and single-cell PDO	Pilot; biopsy quality; poorer success in diffuse/signet-ring; CLIA needed
Schmäche 2024 [86]	Endoscopic biopsies, treatment-naïve	FLOT and components (5-FU, oxaliplatin, docetaxel)	9/73 identified as normal; contamination suspected in 1 misclassified case	Before therapy (treatment-naive); neoadjuvant FLOT	NR	PDOs reflected diverse responses to single drugs	Small sample; delivery > 24 h detrimental; passage-dependent drift; threshold set on exploratory data; turnaround estimated retrospectively; 39% of cultures not expandable (Heidelberg 63% vs. Dresden 24%)
Huang 2026 [87]	Surgical resection or biopsy (mixed); one set from a liver-metastasis biopsy	5-FU, oxaliplatin, SN-38, epirubicin, paclitaxel (S-1 not tested)	Not reported (PDO vs. parental tumor compared by WES, IHC, TMB/MSI)	Mixed: some pts had neoadjuvant therapy before surgery (e.g., GC-012); liver-metastasis PDOs after several lines (GC-008); PDO results not reported to have guided treatment	NR	One sample per tumor; marked inter-PDO heterogeneity; authors call for multi-region biopsies	Small n; short follow-up (~19–21 months; 2 events); mixed settings (adjuvant, neoadjuvant, stage IV); 5-FU used although most pts received S-1 + oxaliplatin; sensitivity defined by IR ≥50% at plasma Css; single center 2 of 13 matched pts received no postoperative therapy

Evidence level: biological fidelity (molecular/phenotypic characterization without clinical comparison); retrospective clinical association (outcomes compared after treatment was already given); prospective clinical concordance (organoids tested alongside prospectively treated patients, but results did not influence treatment); prospective interventional clinical utility (organoid results influence treatment and benefit is demonstrated; none of the listed studies). NR (not reported): information or metrics not reported by the original authors in the primary publication or its Supplementary Materials. Establishment, expansion, drug-testing, and clinically evaluable-pair rates are reported with explicitly stated denominators whenever available; when the relevant denominator was not reported in the primary study, this is indicated as “denominator not reported.” Pts, patients; PDO, patient-derived organoid; Sens, sensitivity; Spec, specificity; PPV, positive predictive value; NPV, negative predictive value; DFS, disease-free survival; PFS, progression-free survival; SRCC, signet-ring cell carcinoma; FLOT, 5-fluorouracil/leucovorin/oxaliplatin/docetaxel; mGOC, metastatic gastro-oesophageal cancer; GEJ, gastroesophageal junction; GC, gastric cancer. NA, not applicable; NR, not reported in the primary study or its Supplementary Materials; WES, whole-exome sequencing; IHC, immunohistochemistry; EOX, epirubicin/oxaliplatin/5-FU; FLOT, 5-FU/leucovorin/oxaliplatin/docetaxel; CLIA, Clinical Laboratory Improvement Amendments.

## Data Availability

No new data were created or analyzed in this study. Data sharing is not applicable to this article.

## Abbreviations

The following abbreviations are used in this manuscript:

2D	Two-dimensional
3D	Three-dimensional
3DGO	Three-dimensional gastric organoid
2DMGO	Two-dimensional monolayer gastric organoid
ACRG	Asian Cancer Research Group
ALI	Air–liquid interface
APC	Antigen-presenting cell
ASC	Adult stem cell
ATP	Adenosine triphosphate
AUC	Area under the curve
BMP	Bone morphogenetic protein
CAF	Cancer-associated fibroblast
CagA	Cytotoxin-associated gene A
CCS	Condensed cellular spheroid
*CDH1*	Cadherin 1 (E-cadherin)
CFU	Colony-forming unit
CIN	Chromosomal instability
CLIA	Clinical Laboratory Improvement Amendments
CPS	Combined positive score
CRISPR	Clustered regularly interspaced short palindromic repeats
CTL	Cytotoxic T lymphocyte
DC	Dendritic cell
DE	Definitive endoderm
DFS	Disease-free survival
DGC	Diffuse-type gastric cancer
DKK1	Dickkopf-related protein 1
DMPP	Dimethylphenylpiperazinium
ECM	Extracellular matrix
EFS	Electrical field stimulation
EGD	Esophagogastroduodenoscopy
EGF	Epidermal growth factor
EHS	Engelbreth–Holm–Swarm
EM	Electron microscopy
EMT	Epithelial–mesenchymal transition
ENCC	Enteric neural crest cell
ENS	Enteric nervous system
EOX	Epirubicin, oxaliplatin, and 5-fluorouracil
ESC	Embryonic stem cell
FD	Functional dyspepsia
FGF	Fibroblast growth factor
FGFR	Fibroblast growth factor receptor
FLOT	5-Fluorouracil, leucovorin, oxaliplatin, and docetaxel
GC	Gastric cancer
GEJ	Gastroesophageal junction
GIST	Gastrointestinal stromal tumor
GMP	Good manufacturing practice
GS	Genomically stable
hAGO	Human antral gastric organoid
HDGC	Hereditary diffuse gastric cancer
hESC	Human embryonic stem cell
hFGO	Human fundic gastric organoid
HGF	Hepatocyte growth factor
HIO	Human intestinal organoid
hiPSC	Human induced pluripotent stem cell
HLA	Human leukocyte antigen
hPSC	Human pluripotent stem cell
huTGO	Human gastric tumor organoid
IC50	Half-maximal inhibitory concentration
ICC	Interstitial cell of Cajal
ICI	Immune checkpoint inhibitor
IF	Immunofluorescence
IGC	Intestinal-type gastric cancer
IM	Intestinal metaplasia
iPSC	Induced pluripotent stem cell
L-NAME	N(ω)-nitro-L-arginine methyl ester
MDSC	Myeloid-derived suppressor cell
MEK	Mitogen-activated protein kinase kinase
mESC	Mouse embryonic stem cell
MSC	Mesenchymal stem cell
MSI	Microsatellite instability
MSI-H	Microsatellite instability-high
MSS	Microsatellite stable
NPV	Negative predictive value
NR	Not reported
PBMC	Peripheral blood mononuclear cell
PCC	Poorly cohesive carcinoma
PDMS	Polydimethylsiloxane
PDO	Patient-derived organoid
PEG	Polyethylene glycol
PFS	Progression-free survival
PMGC	Peritoneal metastatic gastric cancer
PPV	Positive predictive value
PSC	Pluripotent stem cell
QC	Quality control
RA	Retinoic acid
RNF43	Ring finger protein 43
scRNA-seq	Single-cell RNA sequencing
SFEBq	Serum-free floating culture of embryoid body-like aggregates with quick reaggregation
SHH	Sonic hedgehog
SMC	Smooth muscle cell
SRCC	Signet-ring cell carcinoma
STR	Short tandem repeat
T4SS	Type IV secretion system
TCGA	The Cancer Genome Atlas
TEER	Transepithelial electrical resistance
thAGO	Three-germ-layer human antral gastric organoid
thFGO	Three-germ-layer human fundic gastric organoid
TGF-β	Transforming growth factor-beta
TMB	Tumor mutational burden
TME	Tumor microenvironment
TTX	Tetrodotoxin
WES	Whole-exome sequencing ZNRF3 Zinc and ring finger 3
ZNRF3	Zinc and ring finger 3
